# Supramolecular scaffold–directed two-dimensional assembly of pentacene into a configuration to facilitate singlet fission

**DOI:** 10.1126/sciadv.adn7763

**Published:** 2024-09-13

**Authors:** Masato Fukumitsu, Tomoya Fukui, Yoshiaki Shoji, Takashi Kajitani, Ramsha Khan, Nikolai V. Tkachenko, Hayato Sakai, Taku Hasobe, Takanori Fukushima

**Affiliations:** ^1^ Laboratory for Chemistry and Life Science, Institute of Innovative Research, Tokyo Institute of Technology, 4259 Nagatsuta, Midori-ku, Yokohama 226-8501, Japan.; ^2^ Department of Chemical Science and Engineering, School of Materials and Chemical Technology, Tokyo Institute of Technology, 4259 Nagatsuta, Midori-ku, Yokohama 226-8501, Japan.; ^3^ Research Center for Autonomous Systems Materialogy (ASMat), Institute of Innovative Research, Tokyo Institute of Technology, 4259 Nagatsuta, Midori-ku, Yokohama 226-8501, Japan.; ^4^ Open Facility Development Office, Open Facility Center, Tokyo Institute of Technology, 4259 Nagatsuta, Midori-ku, Yokohama 226-8501, Japan.; ^5^ Chemistry and Advanced Material Group, Faculty of Engineering and Natural Sciences, Tampere University, Korkeakoulunkatu 8, FI33720 Tampere, Finland.; ^6^ Department of Chemistry, Faculty of Science and Technology, Keio University, 3-14-1 Hiyoshi, Kohoku-ku, Yokohama, Kanagawa 223-8522, Japan.

## Abstract

Molecular assemblies featuring two-dimensionality have attracted increasing attention, whereas such structures are difficult to construct simply relying on spontaneous molecular assembly. Here, we present two-dimensional assemblies of acene chromophores achieved using a tripodal triptycene supramolecular scaffold, which have been shown to exhibit a strong ability to assemble molecular and polymer motifs two-dimensionally. We designed pentacene and anthracene derivatives sandwiched by two triptycene units. These compounds assemble into expected two-dimensional structures, with the pentacene chromophores having both sufficient overlap to cause singlet fission and space for conformational change to facilitate the dissociation of a triplet pair into free triplets, which is not the case for the anthracene analog. Detailed spectroscopic analysis revealed that the pentacene chromophore in the assembly undergoes singlet fission with a quantum yield of 88 ± 5%, giving rise to triplet pairs, from which free triplets are efficiently generated (Φ_T_ = 130 ± 8.8%). This demonstrates the utility of the triptycene-based scaffold to design functional π-electronic molecular assemblies.

## Teaser

Making both singlet fission and triplet formation of pentacene efficient by spatial arrangement using a supramolecular scaffold.

## INTRODUCTION

Two-dimensional (2D) assemblies consisting of π-electronic systems have attracted increasing attention in a wide range of areas, including supramolecular chemistry, materials science, and organic electronics (*1*–*5*), because of their potential to exhibit particular properties arising from the dimensionality, as well as their morphological compatibility with the architecture of thin-film devices. However, it is rare for molecules to spontaneously assemble into 2D structures. The construction of 2D molecular assemblies has mostly been achieved using tailored design for each building block and/or specific fabrication techniques that require dedicated experimental setups (*6*–*20*). We have proposed an approach based on supramolecular scaffolds (*21*) to rationally construct 2D assemblies with diverse molecules. Supramolecular scaffolds represent molecular building blocks that have the robust ability to form a desired assembly structure upon chemical modifications with various functional groups.

**Fig. 1.**
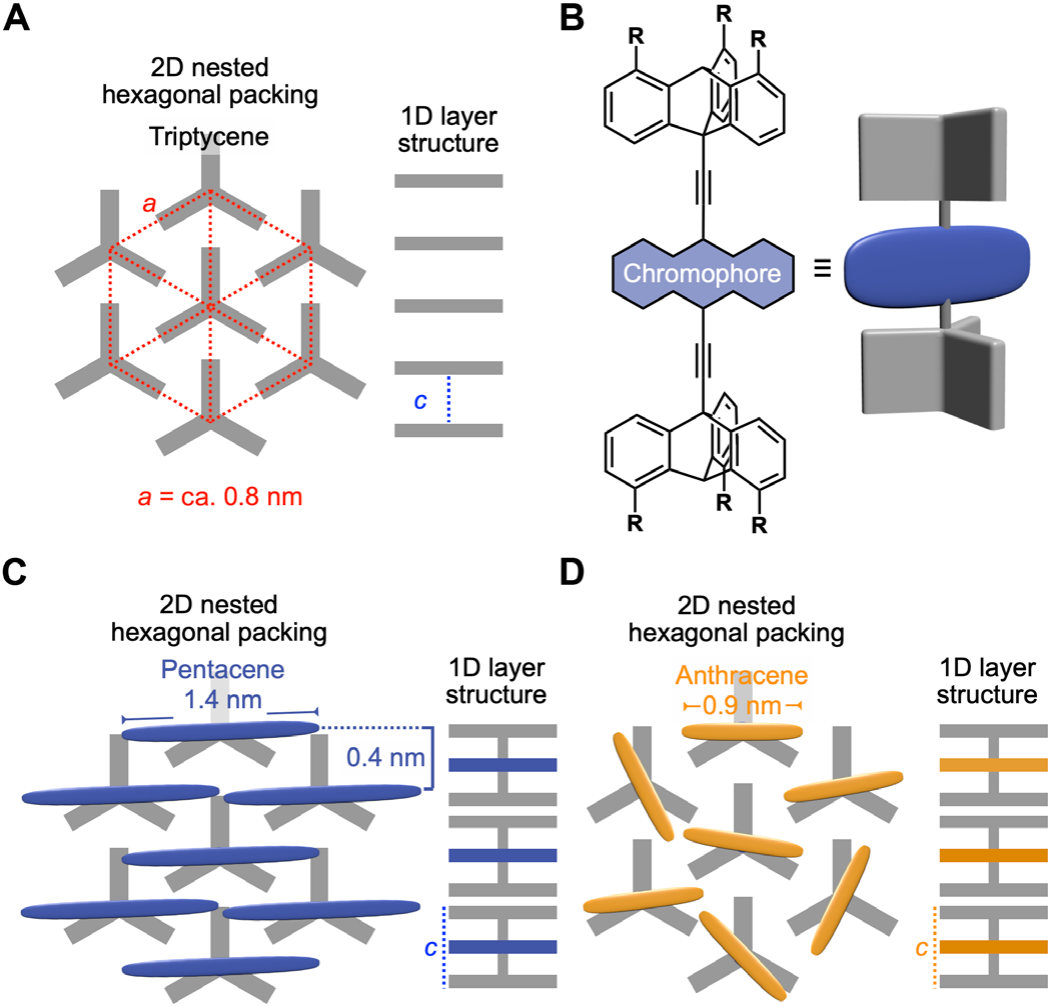
Schematic illustrations of supramolecular-scaffold-directed 2D assembly. (**A**) A typical 2D + 1D structure of self-assembled tripodal triptycene derivatives, (**B**) a tripodal triptycene–based sandwich-type molecule in which the bridgehead positions are connected with an acene chromophore (pentacene or anthracene), and proposed assembly structures formed from (**C**) pentacene- and (**D**) anthracene-containing derivatives. The lengths for the longer axis of anthracene and pentacene are estimated to be 0.92 and 1.40 nm, respectively. On the basis of the PXRD patterns of the assemblies of acene-containing derivatives (Fig. 3, A and B), the pentacene units are considered to be slightly tilted with respect to the 2D triptycene lattice, while the anthracene units are randomly oriented in the 2D plane.

We have demonstrated that 1,8,13-substituted triptycenes (*22*) serve as an excellent supramolecular scaffold to achieve 2D assemblies with a wide variety of molecular units and even polymers (*23*–*29*). This type of tripodal triptycene forms nested hexagonal packing to fill the free volume around the phenylene blades, resulting in highly ordered 2D sheets, which align one-dimensionally (1D) to form a layer structure, referred to as “2D + 1D” structure hereafter (Fig. 1A). In the 2D sheets, the bridgehead positions of triptycene align hexagonally with approximately 8-Å intervals, thus allowing for the construction of a well-defined 2D assembly of fullerene (C_60_), which shows anisotropic charge-carrier transport properties (*24*).

Here, an issue may arise. Although C_60_ features a spherical shape compatible with the geometric features of the 2D triptycene arrays, is it possible to achieve 2D assembly for other π-electronic systems, which are generally planar and anisotropic in structure? This is because void spaces may form when planar molecular units are assembled two dimensionally using the triptycene-based supramolecular scaffold. To address this issue, as well as to confirm the versatility of the approach of creating 2D assemblies using tripodal triptycenes, we examined systems having acene units, such as anthracene and pentacene. We also expected that if these could be assembled two-dimensionally, a molecular arrangement to facilitate singlet fission (SF) (*30*) would be realized.

SF is a phenomenon, in which one singlet excited state (S_1_) is split into a triplet pair [(T_1_T_1_)*] (*30*), and has attracted considerable attention for improving the efficiency of solar cells and optoelectronic devices (*31*–*34*). As exemplified by pentacene derivatives, acenes hold promise as the components of efficient SF materials (*30*, *35*–*44*). However, to realize efficient SF in the solid state, two requirements should be satisfied. First, acene chromophores need to be placed in close proximity to each other for sufficient electronic coupling (*30*, *44*–*50*). Second, the environment around the chromophores needs to be designed to allow them to undergo conformational changes (*30*, *37*, *44*–*53*), so that the orbital overlap within (T_1_T_1_)* is reduced, suppressing unfavorable T_1_T_1_ annihilation and facilitating the dissociation of (T_1_T_1_)* into two free triplets. In view of their geometrical features, 2D assemblies of acenes induced by tripodal triptycenes would be interesting as a motif for efficient SF materials.

**Fig. 2.**
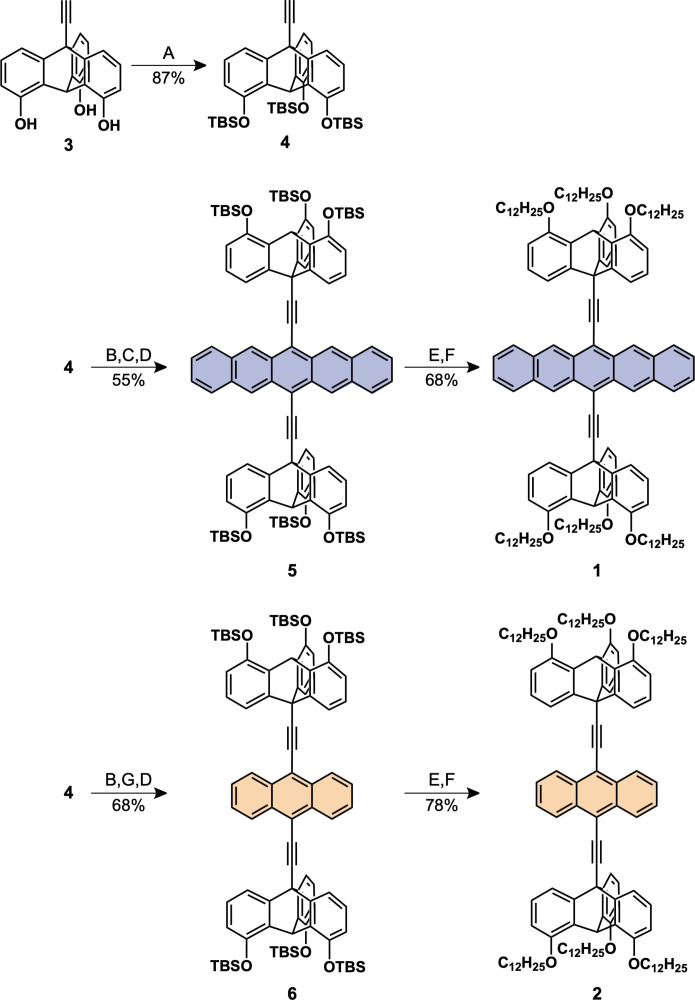
Synthesis of 1 and 2. Reagents and conditions: (**A**) TBSCl, imidazole, DMF, 90°C; (**B**) *n*-BuLi, THF, 0°C; (**C**) 6,13-pentacenedione, 40°C; (**D**) SnCl_2_•2H_2_O, HCl, 25°C; (**E**) TBAF, THF, 25°C; (**F**) K_2_CO_3_, 1-bromododecane, DMF, 80°C; and (**G**) anthraquinone, 40°C.

On the basis of the above consideration, we designed sandwich-type molecules **1** and **2** (Figs. 1B and 2), so that strong π-stacking of the acene chromophores can be avoided, leading to a bilayer structure. **1** and **2** were found to self-assemble into the desired structures (Fig. 1, C and D), where 2D arrays of the acene chromophores are incorporated between the 1D layers. Detailed spectroscopic analysis revealed that, when self-assembled, **1** shows generation of a triplet pair [(T_1_T_1_)*] from one singlet excited state (S_1_) with a quantum yield (Φ_SF_) of 88 ± 5% and a rate constant (*k*_SF_) of 5.9 × 10^12^ s^−1^, which are comparable to those reported for particular types of single-crystalline pentacene derivatives (*49*, *50*). Furthermore, (T_1_T_1_)* dissociates into two free triplets (T_1_ + T_1_) with a quantum yield (Φ_T_) of 130 ± 8.8% and its efficiency (Φ_Diss_) of 74%. In contrast, no SF was observed for the assembly of **2**, where the anthracene chromophores have weak electronic interactions. Here, we report the synthesis, assembled structures, electronic properties, and excited state dynamics of **1** and **2**.

## RESULTS

### Synthesis and characterization

Compounds **1** and **2** were synthesized according to Fig. 2. Hydroxy groups of 1,8,13-trihydroxy-10-ethynyltriptycene **3** (*24*) were protected with a *tert*-butyl-dimethylsilyl (TBS) group to give **4** in 87% yield. The alkyne terminal of **4** was lithiated using *n*-butyl lithium (*n*-BuLi) and then reacted with 6,13-pentacenedione, and the resultant product was treated with SnCl_2_, affording **5** in 55% yield. After the TBS groups of **5** were removed using tetra-*n*-butylammonium fluoride (TBAF), the phenol groups generated were alkylated with 1-bromododecane in *N*,*N*′-dimethylformamide (DMF) in the presence of K_2_CO_3_ to afford **1** in 68% yield. Compound **2** was also synthesized by a procedure similar to that for **1**, except that anthraquinone was used instead of 6,13-pentacenedione. The chemical compositions of **1**, **2**, and their synthetic intermediates were unambiguously characterized by ^1^H and ^13^C nuclear magnetic resonance (NMR), infrared (IR) spectroscopy and atmospheric pressure chemical ionization (APCI) time-of-flight (TOF) mass spectrometry (figs. S1 to S20).

### Thermal properties

The thermal stabilities of **1** and **2** were evaluated based on thermogravimetric analysis (TGA), differential scanning calorimetry (DSC), and ^1^H NMR spectroscopy. In TGA, the 5%-weight loss temperatures of **1** and **2** were 419°C and 404°C, respectively (fig. S21). However, on the basis of ^1^H NMR spectroscopic analysis using thermally treated samples, **1** is thermally stable up to 190°C, while it undergoes partial decomposition at temperatures higher than 200°C (fig. S22). In DSC, **1** did not exhibit any phase transition features in the temperature range of 25° to 200°C (fig. S23). In contrast, **2** is stable upon heating up to at least 270°C, as confirmed by ^1^H NMR spectroscopy of its thermally treated samples (fig. S24). The DSC profile of **2** is reversible even in the third heating/cooling cycle and involves two sets of endothermic/exothermic peaks at 218/197°C and 244/239°C associated with small and large enthalpy changes, respectively (fig. S23). According to optical microscopy, **2** remains solid even when heated to 230°C and shows no signs of the emergence of a liquid crystalline mesophase. As revealed by powder x-ray diffraction (PXRD) experiments (vide infra), the two sets of endothermic/exothermic DSC peaks observed for **2** are due to the presence of polymorphic assemblies with different melting and crystallization temperatures, rather than a two-step sequential phase transition (fig. S25).

### Self-assembled structures

**Fig. 3.**
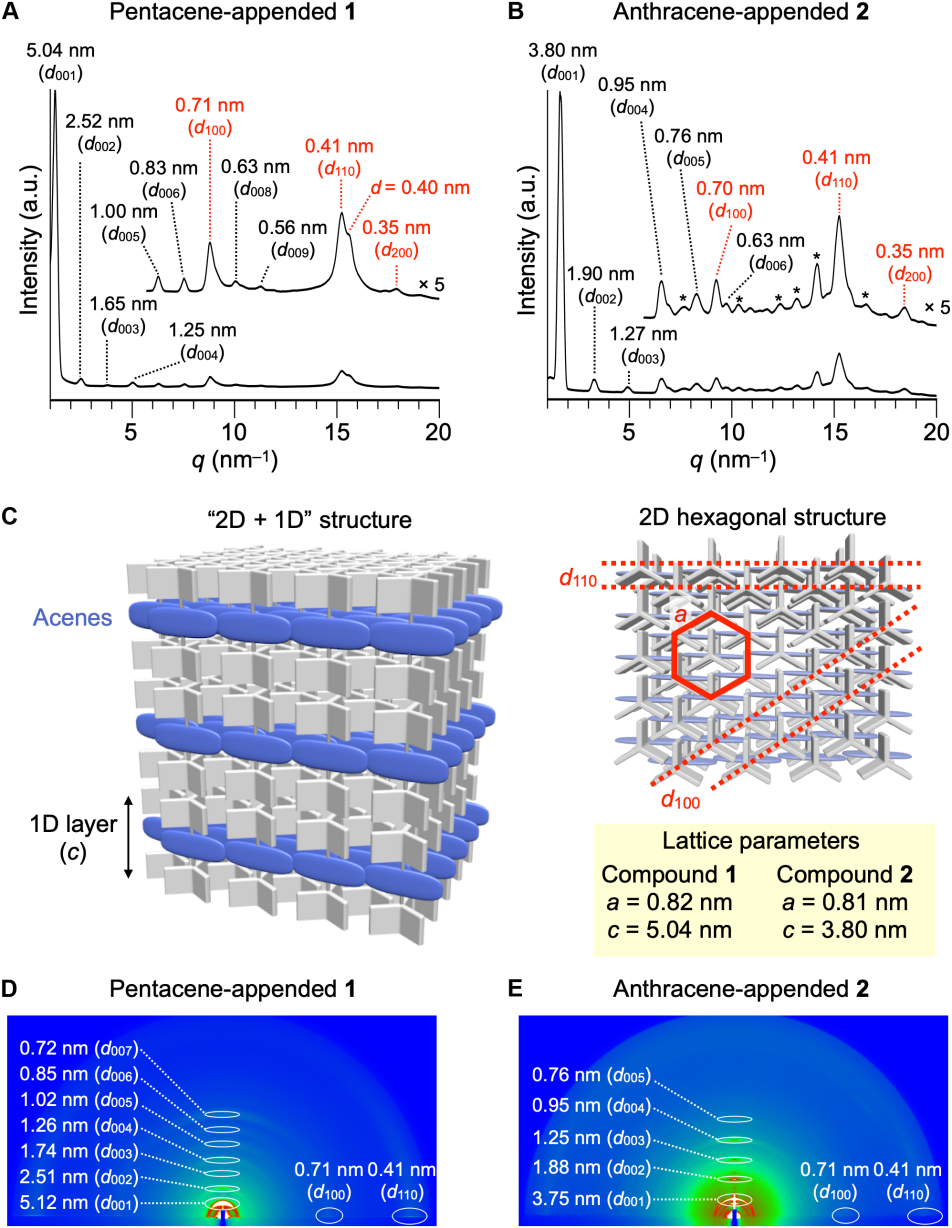
Structural characterization. PXRD patterns of bulk solid samples of (**A**) **1** and (**B**) **2** in a glass capillary (diameter = 1.5 mm) at 25°C. For (A), diffractions from the (007) plane overlap with those from the (100) planes of the hexagonal structure. (**C**) Schematic illustration of a “2D hexagonal + 1D layer” structure of the assemblies of **1** and **2**. GI-XRD images of films of (**D**) **1** and (**E**) **2**, prepared by drop-casting a chloroform solution of **1** or **2** (1 mM) onto a quartz substrate, followed by thermal annealing (50°C, 1 hour) under an atmosphere of a chloroform vapor.

Figure 3 shows PXRD patterns of bulk samples of **1** and **2** at 25°C. A bulk sample of **1** was thermally treated at 50°C for 1 hour under an atmosphere of a chloroform vapor, so as to facilitate the formation of a thermodynamically stable assembly structure. We found that pentacene-appended **1** can form a 2D + 1D structure (Fig. 3C), typical of tripodal triptycenes (*22*, *24*, *28*). Thus, the eight peaks with *d*-spacings of 5.04, 2.52, 1.65, 1.25, 1.00, 0.83, 0.63, and 0.56 nm were assigned to diffractions from the (001), (002), (003), (004), (005), (006), (008), and (009) planes, respectively, of a 1D layer structure (Fig. 3A). The observed 1D layer spacing (*c* = 5.04 nm) is in excellent agreement with the length of the longer molecular axis of **1** (ca. 5.0 nm) (fig. S26). The three peaks observed in the wider-angle region (scattering vector *q* > 8 nm^−1^) have *d*-spacings of 0.71, 0.41, and 0.35 nm with a reciprocal ratio of 1:√3:2. These peaks correspond to diffractions from the (100), (110), and (200) planes of a 2D hexagonal structure with a lattice parameter (*a*) of 0.82 nm, formed by nested packing of the triptycene units. These PXRD features indicate that 2D ordering of the pentacene chromophore can be achieved even though the molecular length (ca. 1.4 nm) is larger than the diameter of the triptycene scaffold (ca. 1.0 nm).

Meanwhile, the PXRD pattern of a bulk solid sample of anthracene-appended **2** (Fig. 3B), measured after being heated once to 270°C to its isotropic hotmelt and then cooled to 25°C at a rate of 10°C/min, showed a set of major diffractions arising from a 2D + 1D structure, together with minor ill-defined diffraction peaks denoted using asterisks. The lattice parameter (*a* = 0.81 nm) of the 2D hexagonal structure is almost identical to that observed for the assembly of **1**. On the other hand, the 1D layer spacing (*c* = 3.80 nm) of assembled **2** is shorter than the length of the longer molecular axis of **2** (ca. 5.0 nm), suggesting that the dodecyloxy chains assemble with a tilted geometry (fig. S26). The PXRD pattern is unchanged even when a bulk sample of **2** was thermally annealed in the presence of a chloroform vapor similar to the case of **1**. Variable temperature–PXRD experiments revealed that the ill-defined diffractions are due to polymorphism involving a structure different from that characterized by the 2D + 1D assembly. As shown in fig. S25, the peak intensities of the minor ill-defined diffractions simultaneously decrease at 230°C, while the peaks of diffractions arising from the 2D + 1D assembly remain intact at this temperature and disappear upon further heating to 260°C. This observation is consistent with the behavior of DSC in the heating process of **2** (fig. S23) and indicates that anthracene-appended **2** in the solid state generates independent structures with different thermal stabilities.

A difference between the PXRD patterns of **1** and **2** should be noted: in the assembly of **1**, a clear shoulder peak can be seen in the slightly wider-angle region of the diffraction peak from the (110) plane. Tripodal triptycenes, when assembled into a 2D + 1D structure, often show diffraction from the (111) plane in a similar region. However, the *d*-spacing (0.40 nm) of this peak is not consistent with the lattice structure with *a* = 0.82 nm and *c* = 5.04 nm in the 2D + 1D assembly of **1**, but rather, it can be rationally explained by considering a stacking structure of the pentacene chromophores as shown in Fig. 1C. It is also reasonable that such a packing structure would not be expected for the assembly of **2** having an anthracene chromophore with a shorter molecular length (Fig. 1D).

### Structural characterization of cast films

We confirmed that compounds **1** and **2**, upon solution casting onto a quartz substrate, form self-assembled structures that are virtually identical to those formed in their bulk solids. Figure 3 (D and E) shows grazing incidence (GI)–XRD profiles of films of **1** and **2**, prepared by drop-casting a chloroform solution of **1** or **2** (1 mM) onto a quartz substrate, followed by thermal annealing (50°C, 1 hour) under an atmosphere of a chloroform vapor. In both cast films, diffraction spots arising from the (00*n*) planes of a 1D layer structure were clearly observed in the meridional direction. Given the fact that higher-order diffractions can be detected for **1**, the self-assembly of **1** has a higher structural order than that of **2**. The two spots with *d*-spacings of 0.71 and 0.41 nm observed in the equatorial direction (Fig. 3, D and E) are due to diffractions from the (100) and (110) planes of a 2D hexagonal structure with a lattice parameter (*a*) of 0.82 nm. Thus, the 2D + 1D structures of assembled **1** and **2** on a quartz substrate are highly oriented in a manner where the 2D sheets formed by nested packing of the triptycene units are parallel to the substrate surface. Note that, unlike in the case of the bulk solid of **2** (Fig. 3B), **2** upon solution casting does not give rise to a polymorph that results in minor diffractions and exclusively forms the 2D + 1D structure.

### Steady-state absorption and emission properties

**Fig. 4.**
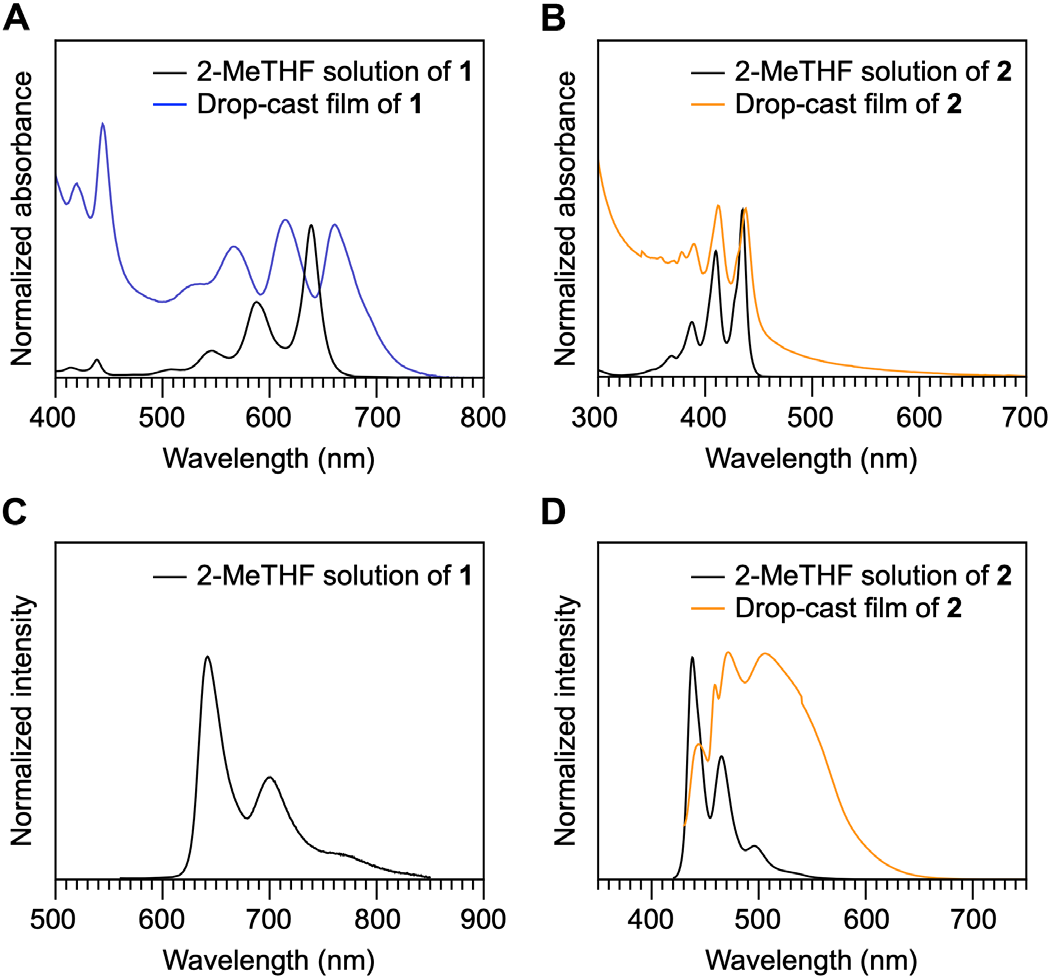
Steady-state absorption and emission spectra. Absorption spectra of 2-MeTHF solutions and drop-cast films on a quartz substrate of (**A**) **1** (5.0 μM) and (**B**) **2** (10 μM) at 25°C under air. Fluorescence spectra of 2-MeTHF solutions and drop-cast films of (**C**) **1** and (**D**) **2** at 25°C under air. The absorption spectra of **1** at a wavelength region from 300 to 800 nm are shown in fig. S27.

Pentacene-appended **1** in 2-methyltetrahydrofuran (2-MeTHF) at 25°C under air displayed absorption maxima (λ_max_) at 545, 596, and 640 nm (Fig. 4A, black curve). As shown in Fig. 4C (black curve), upon excitation at λ_ex_ = 585 nm, **1** emits fluorescence with emission maxima (λ_FL_) at 642, 706, and 773 nm with a quantum yield (Φ_FL_) of 0.24 and a lifetime (τ_FL_) of 17 ns (fig. S28). Anthracene-appended **2** in 2-MeTHF showed absorption maxima at 374, 390, 410, and 439 nm (Fig. 4B, black curve) and emission maxima at 441, 464, 497, and 532 nm (λ_ex_ = 410 nm, Φ_FL_ = 0.72, and τ_FL_ = 4.4 ns; Fig. 4D, black curve, and fig. S28). Since the absorption and emission behaviors of **1** and **2** are similar to those reported for the corresponding diethynyl-appended acene derivatives (*54*, *55*), the presence of the triptycene scaffolds has no influence on the electronic properties of the acene chromophores in solution.

In a drop-cast film of **1** on a quartz substrate, the absorption bands appear at 564, 612, and 659 nm (Fig. 4A, blue curve), which are red-shifted and broadened compared with those observed in solution. Considering also the fact that **1** in the film displays no fluorescence, the pentacene chromophores existing between 2D triptycene arrays strongly interact with one another in the solid state. In contrast, the positions of the absorption maxima of **2** in a drop-cast film are almost identical to those measured in solution (Fig. 4B). Furthermore, upon excitation at λ_ex_ = 404 nm, a drop-cast film of **2** mainly exhibits a broad excimer emission with a maximum at 512 nm (Φ = 0.06; Fig. 4D) (*56*). Although the monomer fluorescence of **2** in the film can also be seen in the shorter-wavelength region, its intensity is weak.

The above difference in spectroscopic behavior between **1** and **2** can be reasonably interpreted in terms of the difference in the size of the acenes. When incorporated between the 2D triptycene arrays, the pentacene chromophores could be arranged to overlap with adjacent chromophores, but effective overlapping of the smaller anthracene chromophores is unlikely (Fig. 1, C and D). Hence, in the ground state, the pentacene chromophores in assembled **1** electronically interact with one another, but the interactions between the anthracene chromophores in assembled **2**, if any, are weak. In the excited state, both compounds display features that reflect the presence of electronic interactions between adjacent acene chromophores, while the nature of these interactions is considered to be different. The conformation of anthracene chromophores of assembled **2** could change so that they overlap with adjacent chromophores, resulting in excimer emission, along with monomer fluorescence. In contrast, the observed nonradiative nature of the pentacene chromophores in assembled **1** suggests the occurrence of SF in this system.

### Time-resolved spectroscopic analysis

To evaluate the excited-state properties of **1** and **2**, we performed time-resolved spectroscopic measurements at room temperature (ca. 20°C). Femtosecond (fs)–transient absorption (TA) spectra of **1** in 2-MeTHF (5.5 μM) upon laser-pulse excitation at 360 nm showed a singlet-singlet (S-S) absorption band in the wavelength region from 410 to 470 nm (fig. S29), typical for 6,13-diethyl-substituted pentacene derivatives (*54*). Nanosecond (ns)–TA spectroscopy of **1** in the presence of anthracene (0.30 mM) as a triplet photosensitizer (*57*) under otherwise identical conditions gave a TA band at around 500 nm (fig. S30). This can be assigned to TA of the pentacene chromophore in the triplet excited state (T_1_), which is generated through energy transfer from the T_1_ state of anthracene. Likewise, we determined the absorption bands of the singlet excited state (S_1_) and T_1_ state of the anthracene chromophore of **2**, which appeared at wavelength regions from 680 to 730 nm (fig. S31) and from 460 to 500 nm, respectively (fig. S32).

**Fig. 5.**
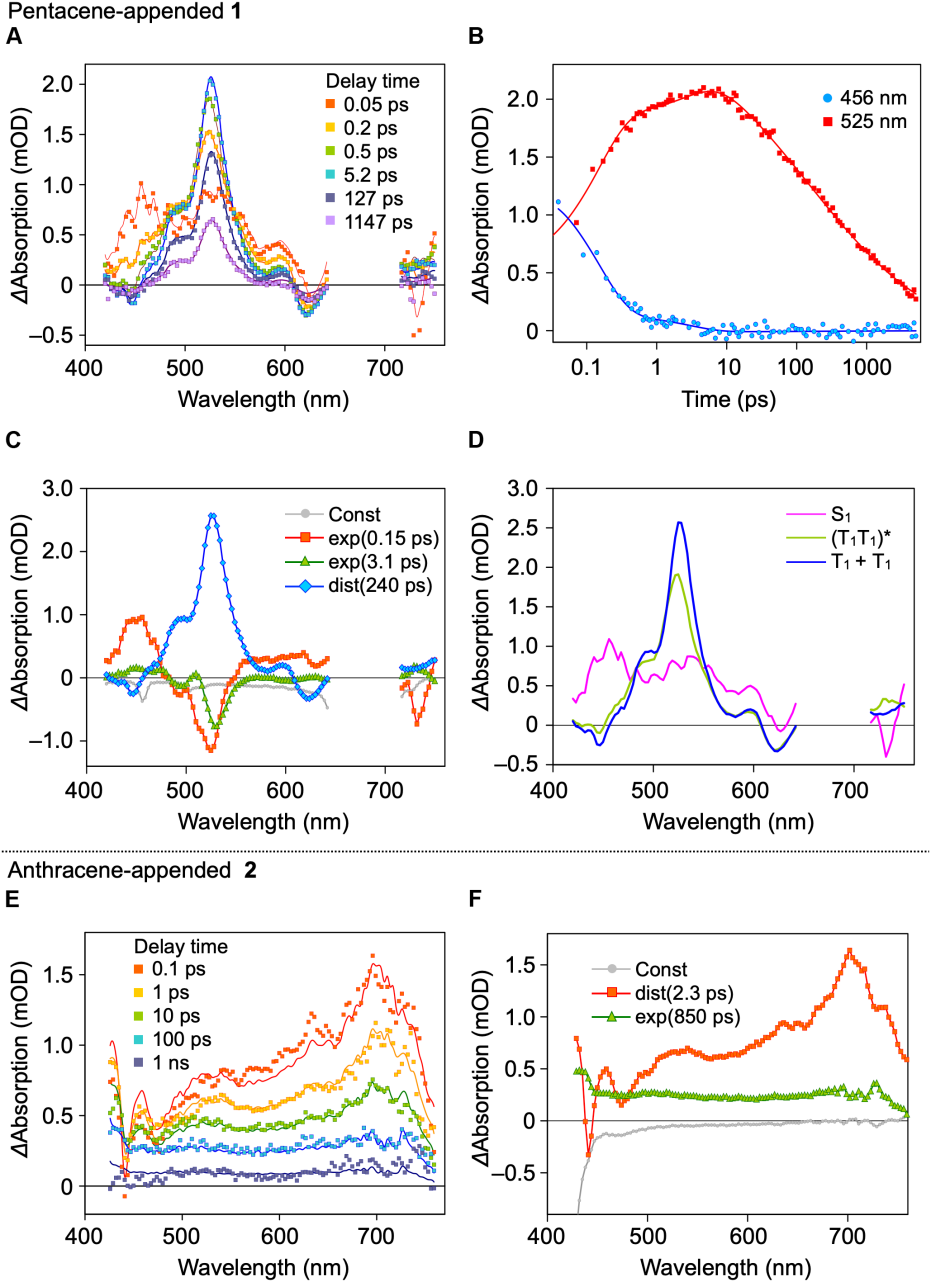
Time-resolved spectroscopic analysis. (**A**) Femtosecond-transient absorption (fs-TA) spectra of a drop-cast film of **1** on a quartz substrate. (**B**) Decay profiles of TA of a drop-cast film of **1** at 456 (blue) and 525 nm (red), observed after laser-pulse excitation at 670 nm. (**C**) Decay-associated spectra (DAS) of **1** obtained from the global fitting analysis. The fitting model used includes two exponential components [exp(0.15 ps) and exp.(3.1 ps)], a distributed decay component [dist(240 ps)], and a response that does not depend on the delay time (Const) due to excitation scattering and other instrumental effects. (**D**) Calculated TA spectra of the intermediate states of **1**. (**E**) fs-TA spectra of a drop-cast film of **2** on a quartz substrate. (**F**) DAS of **2** obtained from the global fitting analysis. The fitting model used includes the distributed decay component [dist(2.3 ps)] attributed to the decay of the singlet excited state, and an exponential decay [exp(850 ps)] from excimer to the ground state. The 2D fs-TA spectra of drop-cast films of **1** and **2** on a quartz substrate are shown in fig. S34.

Figure 5A shows fs-TA spectra of a drop-cast film of **1** on a quartz substrate, measured upon laser-pulse excitation at 670 nm (excitation energy density, ca. 0.1 mJ/cm^2^). At this excitation energy density, the concentration of excitons generated is low, preventing triplet-triplet (T-T) annihilation or any other multiphoton effects (fig. S33). After a delay of 0.05 ps, which is the time-resolution limit in this experimental setup, an S-S absorption band for the pentacene chromophore was seen in the range of 440 to 470 nm (Fig. 5A). The time profile at 456 nm showed a simple decay having a lifetime of ca. 0.2 ps (Fig. 5B). With the disappearance of the S-S absorption, a T-T band for the pentacene chromophore at around 530 nm (*35*–*37*, *54*) rapidly appeared after a delay time of 0.2 ps (Fig. 5A). The observed spectroscopic features indicate that the pentacene chromophores in assembled **1** undergo fast SF. Meanwhile, the time profile corresponding to the T-T band at 525 nm (Fig. 5B) was complex involving multiple intermediate states, which can be accounted for by considering that triplet pairs [(T_1_T_1_)*] generate through SF and subsequently dissociate into two triplets (T_1_ + T_1_) (*44*). The signal at 525 nm continued to grow in the picosecond time domain and then underwent a nonexponential decay in time domain from tens of picoseconds to nanoseconds, during which the TA spectral shape did not change (Fig. 5A).

**Fig. 6.**
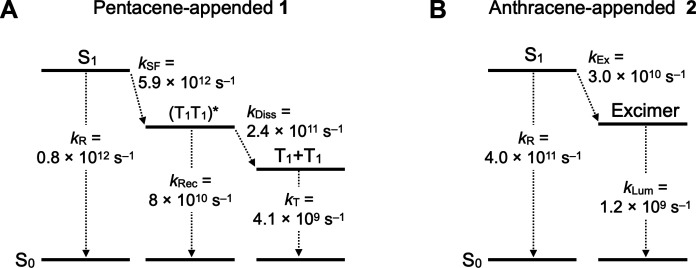
Excited state dynamics. Summary of the rate constant for each process from the S_1_ states of **1** (**A**) and **2** (**B**), where *k*_R_, *k*_SF_, *k*_Rec_, *k*_Diss_, *k*_T_, *k*_Ex_, and *k*_Lum_ are the rate constants for the S_1_ → S_0_ transition, SF, (T_1_T_1_)* → S_0_ transition, dissociation of (T_1_T_1_)* into T_1_ + T_1_, T_1_ → S_0_ transition, excimer formation, and excimer → S_0_ transition, respectively. Because of the time resolution of the instrument, it is difficult to precisely determine rate constants for the SF and S_1_ → S_0_ transition, which are very fast processes. Tables S1 and S2 summarize the lifetimes (τ) for each process.

To gain quantitative insight into the excited state dynamics of the pentacene chromophores in the assembly of **1**, we performed a global fitting analysis of the 2D fs-TA spectra (fig. S34) using three decay-associated components, including a fast subpicosecond exponential component to model S_1_ → (T_1_T_1_)*, a picosecond exponential decay for (T_1_T_1_)* → T_1_ + T_1_, and a distributed decay to model relaxation of the free T_1_ states to the ground state. This fitting model gave a good approximation of the experimental data with a global sigma value of approximately 0.04 milli-optical density (mOD). The obtained decay-associated spectra (DAS) are shown in Fig. 5C. The lifetimes of S_1_ (τ_1_) and (T_1_T_1_)* (τ_2_) were determined to be 0.15 ± 0.01 ps and 3 ± 0.1 ps, respectively. A broad decay distribution of the triplet state was observed in a time range from 5 ps to 10 ns with an average relaxation time of 240 ps (Fig. 5, B and C). Assuming a simple chain of excited state dynamics, S_1_ → (T_1_T_1_)* → T_1_ + T_1_ → S_0_ + S_0_, we calculated the TA spectra of the intermediate species (Fig. 5D). The population of the S_1_ state rapidly decreases with the formation of (T_1_T_1_)* through SF with a quantum yield (Φ_SF_) of 88 ± 5% (theoretical maximum = 100%). Using the kinetic model shown in fig. S35, rate constants for each process were calculated. The quantum yield of the formation of (T_1_T_1_)* is described as Φ_SF_ = *k*_SF_/ (*k*_SF_ + *k*_R_) = 0.88, where *k*_SF_ and *k*_R_ are the rate constants of the formation of (T_1_T_1_)* and the S_1_ → S_0_ transition, respectively. Since the lifetime of S_1_ [τ_1_ = (*k*_SF_ + *k*_R_)^−1^] is 0.15 ps, the individual rate constants are obtained as *k*_SF_ = Φ_SF_ / τ_1_ = 5.9 × 10^12^ s^−1^, and *k*_R_ = τ_1_^−1^ − *k*_SF_ = 0.8 × 10^12^ s^−1^. The rate constant determined for the formation of (T_1_T_1_)* (= *k*_SF_) is comparable to those reported for single-crystalline 6,13-substituted pentacene derivatives (*49*, *50*). Associated with the dissociation of (T_1_T_1_)* with τ_2_ = 3.1 ps, free triplets (T_1_ + T_1_) are formed with a quantum yield (Φ_T_) of 130 ± 8.8% (theoretical maximum = 200%). Considering the quantum yield (Φ_SF_) of 88 ± 5%, the efficiency (Φ_Diss_) of the dissociation of (T_1_T_1_)* into T_1_ + T_1_ is 74% (theoretical maximum = 100%). The rate constants for (T_1_T_1_)* → S_0_ (*k*_Rec_) and (T_1_T_1_)* → T_1_ + T_1_ (*k*_Diss_) are determined to be *k*_Diss_ = Φ_Diss_ / 2τ_2_ = 2.4 × 10^11^ s^−1^ and *k*_Rec_ = τ_2_^−1^ − *k*_Diss_ = 8 × 10^10^ s^−1^, respectively (Fig. 6A). These results indicate that without relying on spontaneous assembly of individual molecules, the triptycene-based supramolecular scaffold can direct the formation of soft molecular assemblies of pentacene, suitable for thin film fabrication, in which fast efficient SF, as well as the generation of two free triplets with moderate efficiency, is realized.

Figure 5E shows the TA spectra of a drop-cast film of **2** on a quartz substrate upon laser-pulse excitation at 400 nm (excitation energy density, ca. 0.3 mJ/cm^2^). After a delay of ca. 0.1 ps, an S-S absorption band of the anthracene chromophore was observed at around 700 nm, similar to in solution (fig. S31). After decay of the S_1_, no T-T absorption band around 480 nm was detected, and a broad absorption band around 700 nm was observed, which may be due to excimer formation (Fig. 4D). Global fitting analysis of the 2D fs-TA spectra (fig. S34) resulted in two DAS (Fig. 5F). The dominant process is the formation of the excimer with an average rate constant of *k*_Ex_ = 3.0 × 10^10^ s^−1^. On the basis of the quantum yield of the excimer emission (Φ = 0.06) obtained for a drop-cast film of **2**, the excimer decays to the ground state with a rate constant of *k*_Lum_ = 1.2 × 10^9^ s^−1^ (Fig. 6B). Consistent with the fact that film samples of **2** did not display T-T absorption (Fig. 5E), the results obtained from the time-resolved spectroscopy confirmed that the anthracene chromophore of the assembly of **2** does not undergo SF. We consider the following two reasons for the absence of SF. First, the SF processes of anthracene derivatives are often endothermic (*58*). On the basis of steady-state fluorescence and phosphorescence (fig. S36) measurements, the anthracene chromophore of **2** has a relationship of *E*(S_1_) (2.82 eV) < 2*E*(T_1_) (2.92 eV). Second, the electronic coupling between the anthracene chromophores of assembled **2** in the ground state is not effective enough to cause SF.

## DISCUSSION

Tripodal triptycene–based supramolecular scaffolds have been demonstrated to strongly promote the assembly of a wide variety of molecular and polymer units into regular “2D hexagonal packing + 1D layer” structures in the bulk solid state or on solid substrates. In this study, we focused on planar π-electronic molecular units to assemble two-dimensionally, with an interest in creating efficient SF systems. Thus, pentacene (**1**) and anthracene derivatives (**2**) sandwiched by two tripodal triptycene units were synthesized and examined in terms of their self-assembly behavior and photophysical properties. Both compounds were found to successfully self-assemble to form 2D + 1D structures. In the assembly of **1**, the pentacene chromophores, which have a size larger than that of the diameter of the triptycene framework, have effective overlap to cause SF, while such an overlap between the chromophores does not occur in the assembly of **2**. Steady-state and time-resolved spectroscopic experiments revealed that the pentacene chromophore in assembled **1** shows fast and efficient SF to generate triplet pairs [(T_1_T_1_)*] with a rate constant of 5.9 × 10^12^ s^−1^ and a quantum yield of 88 ± 5%, respectively. These values are comparable to those observed for particular types of single-crystalline pentacene derivatives (*49*, *50*). Furthermore, the quantum yield for the generation of two free triplets is as high as 130 ± 8.8%. In contrast, the anthracene chromophore in assembled **2** does not exhibit SF, most likely due to weak electronic coupling between the chromophores and/or the relationship between *E*(S_1_) and *E*(T_1_). The successful construction of the pentacene assembly exhibiting both 2D structural order and reasonable SF performance as a thin film, achieved using the triptycene scaffold, may offer advantages over other molecular assemblies in solution and single crystalline forms in terms of device applications. Although we have not yet succeeded in orienting the 2D pentacene layer perpendicular to the substrate surface to fit the current device configuration, configurations with laterally arranged electrodes, such as a comb-shaped electrode, are compatible with this material form and may open the way for device applications. Also important is that the present results demonstrate the great potential of the approach using the triptycene scaffold for the development of functional 2D assemblies with π-conjugated molecular units.

## MATERIALS AND METHODS

### Materials

Unless otherwise stated, all commercial reagents were used as received. 1,8,13-Trihydroxy-10-ethynyltriptycene (**3**) was synthesized according to a previously reported procedure (*24*).

### Methods

Preparative size exclusion chromatography (SEC) was performed on a Japan Analytical Industry LC-9210NEXT recycling preparative HPLC system, equipped with JAIGEL-1HH and JAIGEL-2HH columns and a multiwavelength detector (MD-2010Plus) using CHCl_3_ as an eluent. NMR spectroscopy measurements were carried out on a Bruker model AVANCE-400 spectrometer (400.0 MHz for ^1^H) or a Bruker model AVANCE-500 spectrometer (125.7 MHz for ^13^C). Chemical shifts (δ) are expressed relative to the resonances of the residual nondeuterated solvents for ^1^H [CDCl_3_^1^H(δ) = 7.26 parts per million (ppm) and 1,1,2,2-tetrachloroethane-*d*_2_: ^1^H(δ) = 6.00 ppm] and ^13^C [CDCl_3_^13^C(δ) = 77.0 ppm and 1,1,2,2-tetrachloroethane-*d*_2_: ^13^C(δ) = 74.2 ppm]. Absolute values of the coupling constants are given in hertz, regardless of their sign. Multiplicities are abbreviated as singlet (s), doublet (d), triplet (t), multiplet (m), and broad (br). IR spectra were recorded at 25°C on a JASCO model FT/IR-6600ST Fourier-transform infrared (FT-IR) spectrometer. High-resolution APCI-TOF mass spectrometry measurements were performed on a Bruker model microTOF II mass spectrometer equipped with an APCI probe. TGA was performed on a Shimadzu model TGA-50 thermogravimetric analyzer. DSC measurements were carried out on a Mettler-Toledo model DSC 1 differential scanning calorimeter, where temperature and enthalpy were calibrated with In (430 K, 3.3 J/mol) and Zn (692.7 K, 12 J/mol) standard samples in sealed Al pans. Cooling and heating profiles were recorded and analyzed using the STAR^e^ software system. Electronic absorption spectra were recorded in a quartz cell on a JASCO model V-670 UV/Vis spectrophotometer. Fluorescence spectra were measured in a quartz cell on a JASCO model FP-8500 spectrophotometer. The absolute photoluminescent quantum yields (PLQYs) were measured using a Hamamatsu Photonic model Quantaurus-QYC11347 absolute PLQY spectrometer. Fluorescence lifetimes were measured on a HORIBA model Scientific time-correlated single-photon counting system (FluoroCube) with the laser light (DeltaDiode, laser diode head) as an excitation source. The laser operation wavelength, pulse width, and frequency were 404 nm, 50 ps, and 1 MHz, respectively. The practical time resolution is 15 ps by deconvolution of an observed trace with the analytical software (DAS6).

### XRD analysis

Powder XRD patterns of bulk samples were measured in a glass capillary (diameter = 1.5 mm) using a Rigaku model NANOPIX equipped with a HyPix-6000 (Rigaku) detector. The scattering vector (*q* = 4πsinθ/λ), scattering angle θ, and the position of the incident x-ray beam on the detector were calibrated using several orders of layer reflections from silver behenate (d = 58.380 Å), where λ refers to the wavelength of the x-ray beam (Cu Kα, 1.54 Å). The sample-to-detector distance was ca. 90 mm. Using the Rigaku 2DP software, the obtained diffraction patterns were integrated along the Debye-Scherrer ring to afford 1D intensity data. GI-XRD measurements for film samples were performed on a Rigaku model NANOPIX equipped with a HyPix-6000 (Rigaku) detector, where films were exposed to an incident x-ray beam (Cu Kα, 1.54 Å) with an incident angle of 0.2°.

### Time-resolved spectroscopic measurements

ns-TA spectroscopy measurements were carried out on a Unisoku model TSP-2000 flash spectrometer. A Surelite-I Nd-YAG (Q-switched) laser was employed for the flash photo-irradiation and 150-W Xenon arc and halogen lamps were used as the monitor light source. fs-TA spectroscopy measurements were carried out using a femtosecond pump-probe system. A Libra F laser system (Coherent Inc.) was used to create fundamental light pulses at 800 nm at a repetition rate of 1 kHz. The pulse energy was 1 mJ, and the pulse duration was approximately 100 fs. The fundamental beam was split in two, and the majority of the beam energy (roughly 90%) was directed to a Topas C optical parametric amplifier (Light Conversion Ltd.) to produce excitation pulses at the desired wavelength. The rest of the fundamental beam was delivered to a white continuum generator (sapphire crystal) for sample probing in the range 410 to 750 nm. The probe beam was split in two to record reference and signal responses. The measurement system (ExciPro, CDP Inc.) was equipped with a silicon charge-coupled device array for measurements in the visible part of the spectrum. The measurements were carried out by comparing responses with and without excitation using a chopper synchronized with the fundamental laser pulses. The spectra were typically acquired by recording 5000 shots, i.e., averaging over 5 s. Excitation energy dependence was studied separately and the excitation energy was sufficiently low to exclude multiple exciton effects in the reported data unless otherwise stated. The measured data were fitted globally to obtain DAS and characteristic time constants associated with the decays. To account for nonexponential decays typical for molecular films, a distributed decay model was used, which assumes a Gaussian distribution of the lifetime in logarithmic scale

$$p\left(\tau \right)=\text{exp}\left[\frac{-1}{2b^{2}}{\left(\text{ln}\frac{\tau }{\tau_{0}}\right)}^{2}\right]$$

where *p*(τ) is the probability density to find decay with lifetime (τ), τ_0_ is the average lifetime, and *b* is the relative distribution width, e.g., the most of the decay lifetimes are within the range from τ_0_/*b* to τ_0_*b*.

For a drop-cast film of **1**, an excitation wavelength of 670 nm, commonly used to evaluate the SF properties of pentacene derivatives, was used. For a drop-cast film of **2**, an excitation wavelength of 400 nm was used. The quantum yields for the formations of (T_1_T_1_)* and T_1_ + T_1_ were calculated using their molar absorption coefficients and the concentration of S_1_ obtained from Δabsorption (Fig. 5 and fig. S34).

### Synthesis

#### Compound 4

Under nitrogen at 25°C, *tert*-butyldimethylsilyl chloride (1.47 g, 9.75 mmol) and imidazole (1.31 g, 19.2 mmol) were added to a DMF solution (80 ml) of **3** (260 mg, 0.797 mmol), and the resultant mixture was then stirred at 90°C for 13 hours. After being cooled to 25°C, the reaction mixture was poured into water (15 ml), and extracted with diethyl ether (15 ml, three times). The combined organic extract was washed with water (30 ml) and brine (30 ml), dried over anhydrous MgSO_4_, and evaporated to dryness under reduced pressure. The residue was subjected to column chromatography on SiO_2_ [CH_2_Cl_2_/hexane 1/20 (v/v)] to allow isolation of **4** as a white powder (462 mg, 0.691 mmol) in 87% yield: ^1^H NMR (400 MHz, CDCl_3_, 25°C): δ (ppm) 7.38 (d, *J* = 7.6 Hz, 3H), 6.87 (dd, *J* = 7.6 Hz, *J* = 7.6 Hz, 3H), 6.60 (s, 1H), 6.58 (d, *J* = 7.6 Hz, 3H), 3.20 (s, 1H), 0.99 (s, 27H), and 0.27 (s, 18H). ^13^C NMR (126 MHz, CDCl_3_, 25°C): δ (ppm) 150.4, 147.0, 134.1, 124.9, 117.4, 115.5, 79.9, 79.6, 53.7, 34.1, 26.6, 18.8, and −3.4. FT-IR (KBr): ν (cm^−1^) 3289, 2960, 2930, 2860, 2339, 1598, 1482, 1456, 1425, 1389, 1272, 1200, 1145, 1001, 952, 940, 834, 825, 783, 657, 574, and 482. ACPI-TOF mass: calcd. For C_40_H_56_O_3_Si_3_ [M + H]^+^; mass/charge ratio (*m*/*z*) = 669.3610; found: 669.3616. ^1^H and ^13^C NMR, FT-IR, and ACPI-TOF MS spectra of **4** are shown in figs. S1 to S4, respectively.

#### Compound 5

Under nitrogen at 0°C, a hexane solution of *n*-BuLi (1.6 M, 70 μl, 0.11 mmol) was added dropwise to a THF solution (0.5 ml) of **4** (62.0 mg, 0.093 mmol). After being warmed to 25°C, the resultant solution was stirred for 1 hours, to which 6,13-pentacenedione (12.4 mg, 0.040 mmol) was added. The reaction mixture was stirred at 40°C for 4 hours and then allowed to cool to 25°C. SnCl_2_•2H_2_O (65.0 mg, 0.29 mmol) and an aqueous solution of HCl (6.0 M, 150 μl) were added to the reaction mixture. After being stirred for 2 hours at 25°C, the reaction mixture was poured into water (10 ml) and extracted with ethyl acetate (10 ml, three times). The combined organic extract was washed with water (10 ml) and brine (15 ml), dried over anhydrous MgSO_4_, and evaporated to dryness under reduced pressure. The residue was subjected to recycling SEC (1HH + 2HH, CHCl_3_) to allow isolation of **5** as a purple solid (36 mg, 0.022 mmol) in 55% yield: ^1^H NMR (400 MHz, CDCl_3_, 25°C): δ (ppm) 9.75 (s, 4H), 8.08 (d, *J* = 7.5 Hz, 4H), 7.92 (d, *J* = 7.5 Hz, 6H), 7.43 (d, *J* = 7.5 Hz, 4H), 7.03 (dd, *J* = 7.5 Hz, *J* = 7.5 Hz, 6H), 6.79 (s, 2H), 6.70 (d, *J* = 7.5 Hz, 6H), 1.06 (s, 54H), and 0.35 (s, 36H). ^13^C NMR (126 MHz, CDCl_3_, 25°C): δ (ppm) 150.7, 147.9, 134.5, 132.6, 131.0, 128.8, 126.4, 126.2, 125.2, 118.3, 117.6, 116.0, 100.4, 90.0, 55.9, 26.5, 18.9, and −3.3. FT-IR (KBr): ν (cm^−1^) 3059, 2961, 2930, 2856, 2363, 1600, 1483, 1388, 1269, 1230, 1187, 1130, 1003, 927, 834, 780, 746, 669, 544, and 459. ACPI-TOF mass: calcd. For C_102_H_122_O_6_Si_6_ [M]^+^; *m*/*z* = 1610.7852; found: 1610.7819. ^1^H and ^13^C NMR, FT-IR, and ACPI-TOF MS spectra of **5** are shown in figs. S5 to S8, respectively.

#### Compound 1

Under nitrogen at 25°C, a THF solution of TBAF (1.0 M, 0.56 ml, 0.56 mmol) was added dropwise to a THF solution (0.8 ml) of **5** (30.0 mg, 19.0 μmol), and the resultant mixture was stirred at 25°C for 20 hours. The reaction mixture was poured into an aqueous solution of HCl (1.0 M, 10 ml), and a purple precipitate thus formed was collected by filtration, washed with water, and dried under reduced pressure. Then, the residue was suspended in DMF (1.0 ml) with solid K_2_CO_3_ (121 mg, 0.88 mmol). To the suspension was added a DMF solution (0.5 ml) of 1-bromododecane (0.10 ml, 0.33 mmol), and the resultant mixture was stirred at 80°C for 72 hours. After being allowed to cool to 25°C, the reaction mixture was poured into water (5 ml) and extracted with CHCl_3_ (5 ml, three times). The combined organic extract was washed with water (10 ml) and brine (10 ml), dried over anhydrous MgSO_4_, and evaporated to dryness under reduced pressure. The residue was recrystallized from a mixture of hexane and CHCl_3_ [1/1 (v/v), 10 ml] to give **1** as a purple solid (19 mg, 9.8 μmol) in 68% yield: ^1^H NMR (400 MHz, CDCl_3_, 25°C): δ (ppm) 9.74 (s, 4H), 8.04 (d, *J* = 7.5 Hz, 4H), 7.88 (d, *J* = 7.5 Hz, 6H), 7.43 (d, *J* = 7.5 Hz, 4H), 7.13 (s, 2H), 7.10 (dd, *J* = 7.5 Hz, *J* = 7.5 Hz, 6H), 6.72 (d, *J* = 7.5 Hz, 6H), 4.06 (t, *J* = 6.4 Hz, 12H), 1.97–1.90 (m, 12H), 1.69–1.61 (m, 12H), 1.48–1.24 (m, 96H), and 0.90 (t, *J* = 6.4 Hz, 18H). ^13^C NMR (126 MHz, 1,1,2,2-tetrachloroethane-*d*_2_, 100°C): δ (ppm) 154.9, 148.5, 134.3, 133.2, 131.5, 129.1, 126.9, 126.1, 118.8, 116.3, 111.7, 100.7, 90.3, 69.9, 56.2, 33.5, 32.3, 30.3, 30.2, 30.1, 29.7, 29.5, 26.6, 23.1, and 14.4. FT-IR (KBr): ν (cm^−1^) 3038, 2924, 2851, 2362, 1599, 1466, 1279, 1233, 1131, 1057, 873, 783, 741, 673, 603, 465, and 455. ACPI-TOF mass: calcd. For C_138_H_182_O_6_ [M]^+^; *m*/*z* = 1935.3931; found: 1935.3995. ^1^H and ^13^C NMR, FT-IR, and ACPI-TOF mass spectra of **1** are shown in figs. S9 to S12, respectively.

#### Compound 6

By a procedure similar to **5**, compound **6** (47 mg, 0.026 mmol) was obtained from **4** (56 mg, 0.084 mmol) and anthraquinone (7.9 mg, 0.038 mmol) in 68% yield: ^1^H NMR (400 MHz, CDCl_3_, 25°C): δ (ppm) 9.07 (d, *J* = 8.1, 4H), 7.75 (d, *J* = 8.1 Hz, 4H), 7.72 (d, *J* = 8.1 Hz, 6H), 6.96 (dd, *J* = 8.1 Hz, *J* = 8.1 Hz, 6H), 6.73 (s, 2H), 6.66 (d, *J* = 8.1 Hz, 6H), 1.04 (s, 54H), and 0.32 (s, 36H). ^13^C NMR (126 MHz, CDCl_3_, 25°C) δ (ppm): 150.6, 147.8, 134.4, 132.8, 127.5, 127.3, 125.2, 118.7, 117.6, 115.9, 88.5, 88.8, 55.6, 34.4, 26.6, 18.9, and −3.31. FT-IR (KBr): ν (cm^−1^) 3071, 2957, 2929, 2857, 2341, 1599, 1482, 1457, 1274, 1247, 1038, 1001, 940, 867, 838, 782, 747, 672, and 642. ACPI-TOF mass: calcd. For C_94_H_118_O_6_Si_6_ [M + H]^+^; *m*/*z* = 1511.7617; found: 1511.7705. ^1^H and ^13^C NMR, FT-IR, and ACPI-TOF mass spectra of **6** are shown in figs. S13to S16, respectively.

#### Compound 2

By a procedure similar to **1**, compound **2** (54.8 mg, 29.8 μmol) was obtained from **6** (58 mg, 38 μmol) in 78% yield: ^1^H NMR (400 MHz, CDCl_3_, 25°C): δ (ppm) 9.07 (d, *J* = 7.6 Hz, 4H), 7.74 (d, *J* = 7.6 Hz, 4H), 7.68 (d, *J* = 7.6 Hz, 6H), 7.06 (s, 2H), 7.04 (dd, *J* = 7.6 Hz, *J* = 7.6 Hz, 6H), 6.69 (d, *J* = 7.6 Hz, 6H), 4.03 (t, *J* = 6.4 Hz, 12H), 1.95–1.88 (m, 12H), 1.66–1.59 (m, 12H), 1.45–1.26 (m, 96H), and 0.92 (t, *J* = 6.4 Hz, 18H). ^13^C NMR (126 MHz, CDCl_3_, 25°C): δ (ppm) 154.1, 147.8, 133.3, 132.7, 127.5, 127.2, 125.6, 118.7, 115.5, 110.9, 98.2, 88.5, 69.0, 55.2, 32.0, 29.9, 29.8, 29.7, 29.4, 26.1, 22.7, and 14.1. FT-IR (KBr): ν (cm^−1^) 3074, 2921, 2854, 2366, 1601, 1488, 1397, 1278, 1223, 1151, 1064, 868, 784, 743, and 642. ACPI-TOF mass: calcd. For C_130_H_178_O_6_ [M]^+^; *m*/*z* = 1835.3618; found: 1835.3626. ^1^H and ^13^C NMR, FT-IR, and ACPI-TOF mass spectra of **2** are shown in figs S17 to S20, respectively.
