# Supplementary material for: Supramolecular scaffold–directed two-dimensional assembly of pentacene into a configuration to facilitate singlet fission
Source: Sci Adv. 2024 Sep 13;10(37):eadn7763. doi: 10.1126/sciadv.adn7763 (PMC11397492; doi:10.1126/sciadv.adn7763)
Supplement: Supplementary file 1 — Figs. S1 to S36 Tables S1 and S2 [file sciadv.adn7763_sm.pdf]

Science Advances
AAAS

Supplementary Materials for

Supramolecular scaffold–directed two-dimensional assembly of pentacene into a configuration to facilitate singlet fission

Masato Fukumitsu *et al.*

Corresponding author: Tomoya Fukui, fukui@res.titech.ac.jp; Nikolai V. Tkachenko, nikolai.tkachenko@tuni.fi; Taku Hasobe, hasobe@chem.keio.ac.jp; Takanori Fukushima, fukushima@res.titech.ac.jp

Sci. Adv. **10**, eadn7763 (2024)
DOI: 10.1126/sciadv.adn7763

This PDF file includes:

Figs. S1 to S36
Tables S1 and S2

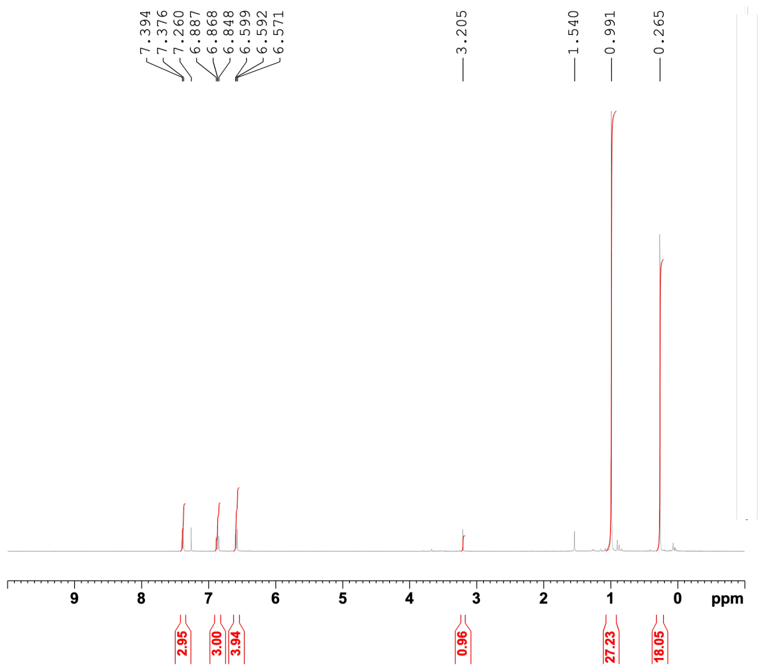

Fig. S1. Characterization of 4. ^{1}H NMR spectrum (400 MHz) of **4** in $CDCl_3$ at 25 °C.

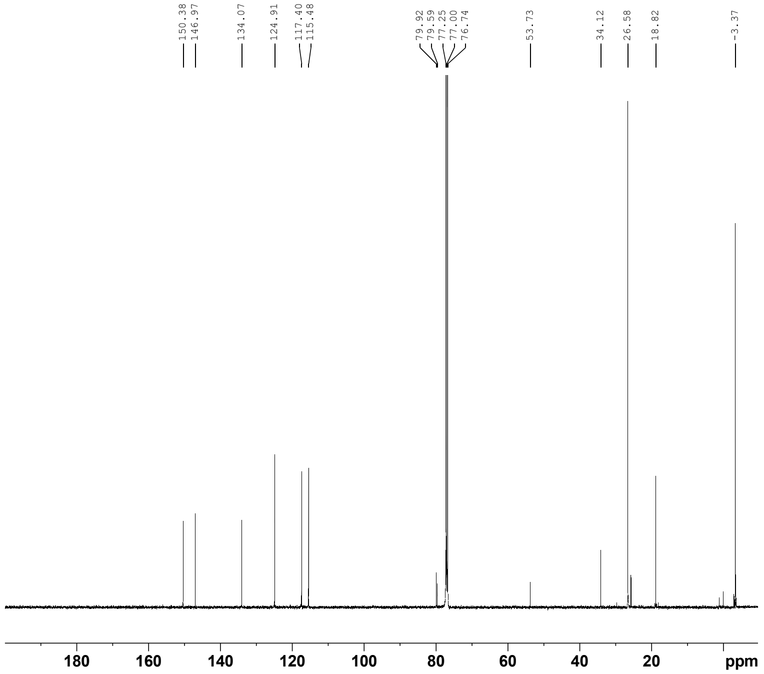

Fig. S2. Characterization of 4. ^{13}C NMR spectrum (126 MHz) of **4** in $CDCl_3$ at 25 °C.

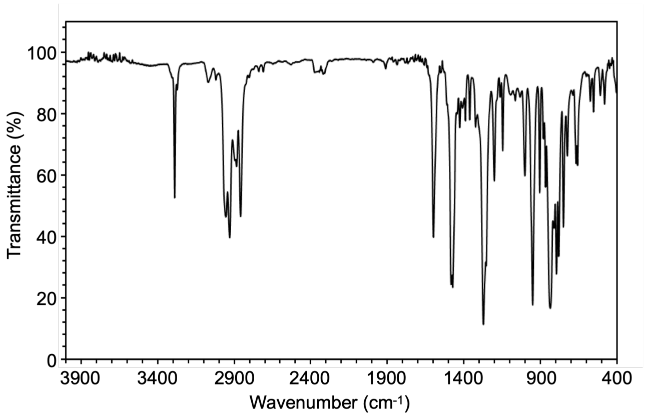

Fig. S3. Characterization of 4. FT-IR spectrum of **4** (KBr) at 25 °C.

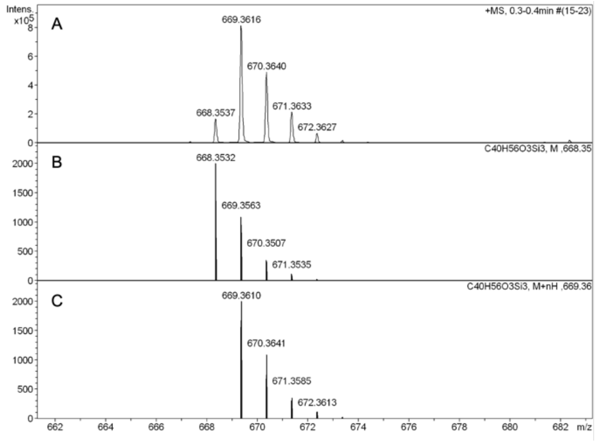

Fig. S4. Characterization of 4. (A) Observed and (B,C) simulated high-resolution APCI-TOF mass spectra of **4**.

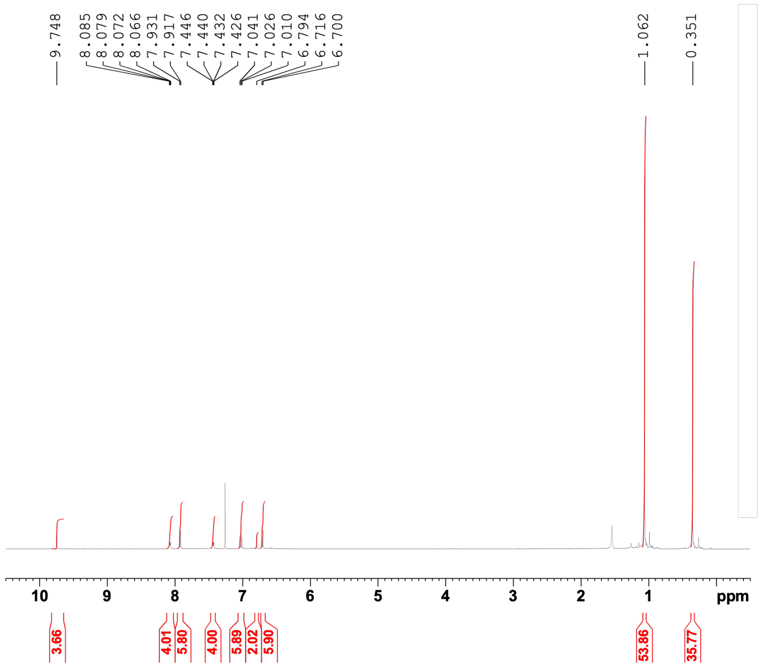

Fig. S5. Characterization of 5. ^{1}H NMR spectrum (400 MHz) of **5** in $CDCl_3$ at 25 °C.

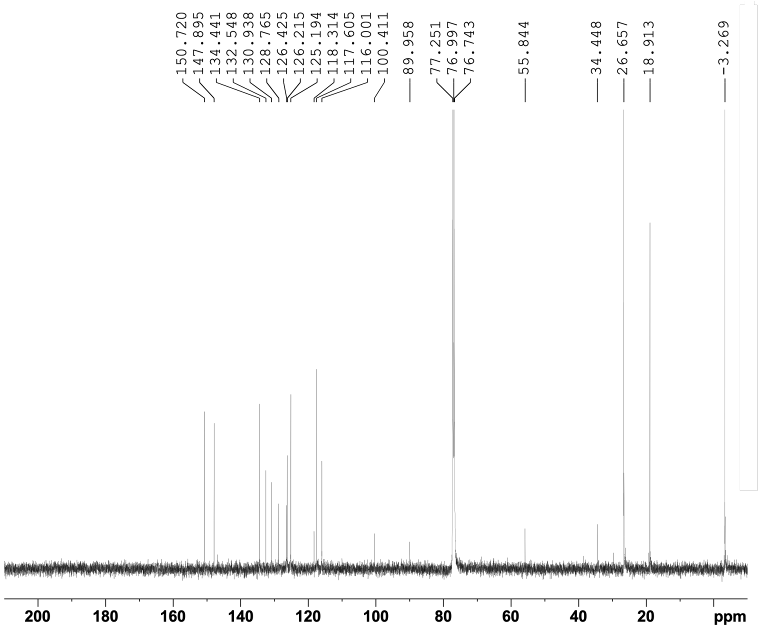

Fig. S6. Characterization of 5. ^{13}C NMR spectrum (126 MHz) of **5** in $CDCl_3$ at 25 °C.

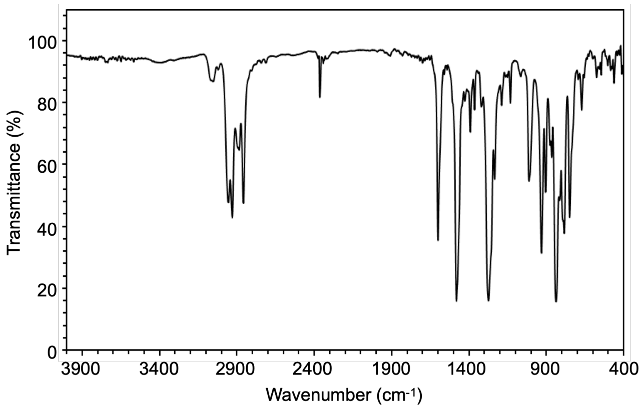

Fig. S7. Characterization of 5. FT-IR spectrum of **5** (KBr) at 25 °C.

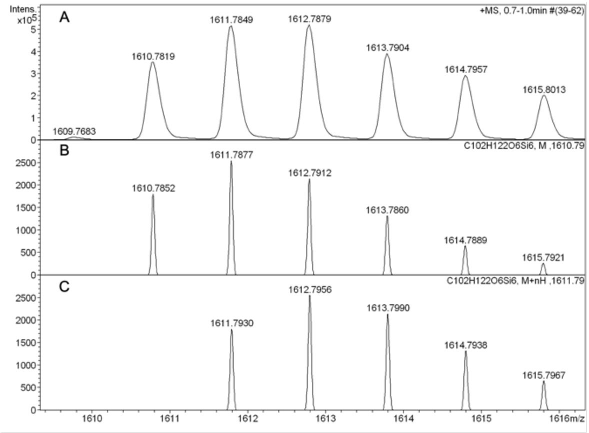

Fig. S8. Characterization of 5. (A) Observed and (B,C) simulated high-resolution APCI-TOF mass spectra of **5**.

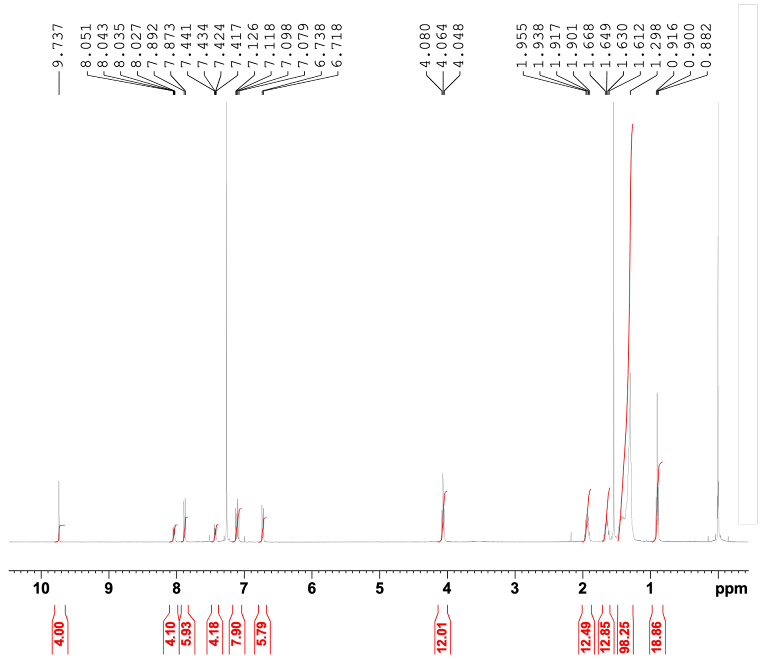

Fig. S9. Characterization of 1. ^{1}H NMR spectrum (400 MHz) of **1** in $CDCl_3$ at 25 °C.

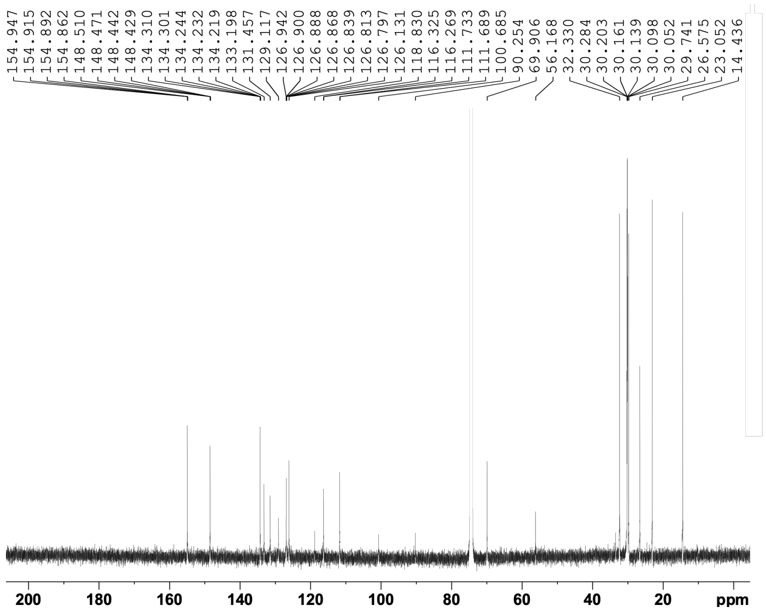

Fig. S10. Characterization of 1. ^{13}C NMR spectrum (126 MHz) of **1** in 1,1,2,2-tetrachloroethane-*d*$_2$ at 100 °C.

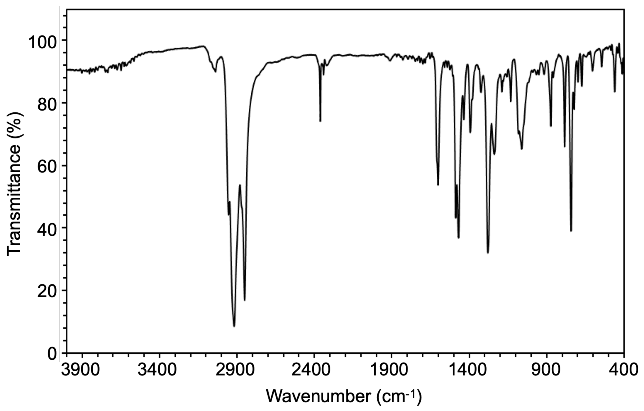

Fig. S11. Characterization of 1. FT-IR spectrum of **1** (KBr) at 25 °C.

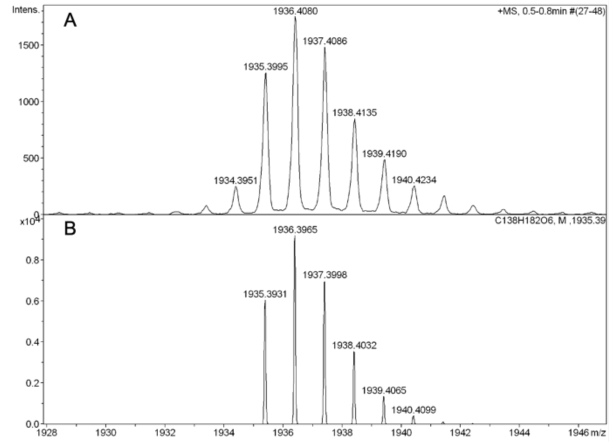

Fig. S12. Characterization of 1. (A) Observed and (B) simulated high-resolution APCI-TOF mass spectra of **1**.

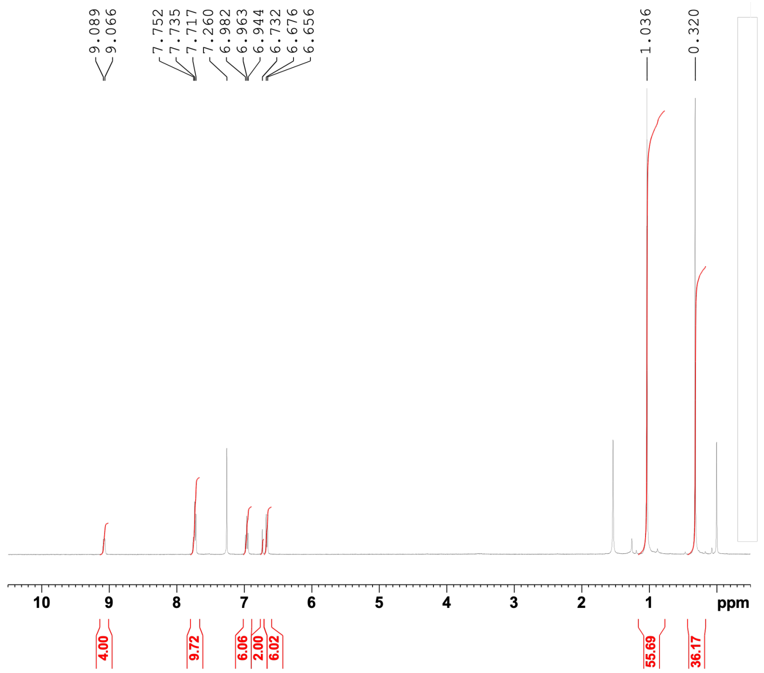

Fig. S13. Characterization of 6. ^{1}H NMR spectrum (400 MHz) of **6** in $CDCl_3$ at 25 °C.

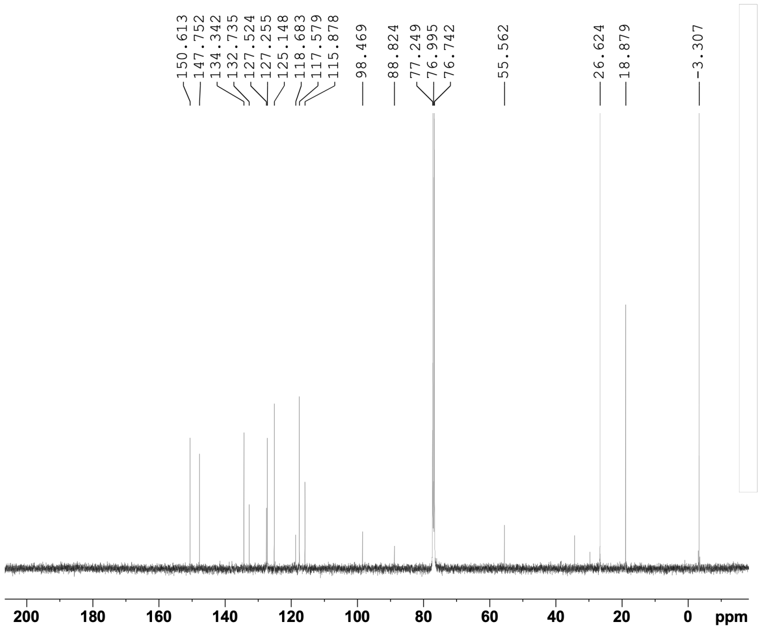

Fig. S14. Characterization of 6. ^{13}C NMR spectrum (126 MHz) of **6** in $CDCl_3$ at 25 °C.

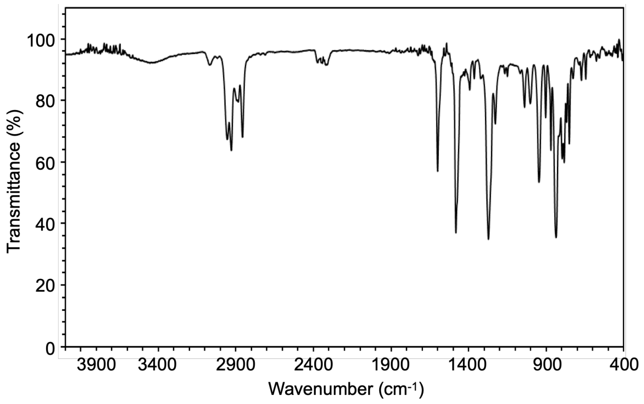

Fig. S15. Characterization of 6. FT-IR spectrum of **6** (KBr) at 25 °C.

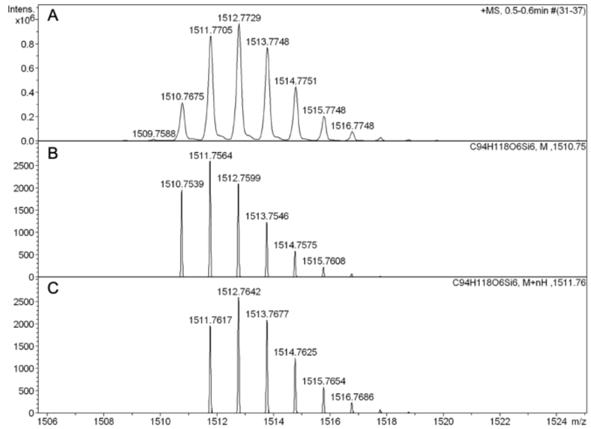

Fig. S16. Characterization of 6. (A) Observed and (B,C) simulated high-resolution APCI-TOF mass spectra of **6**.

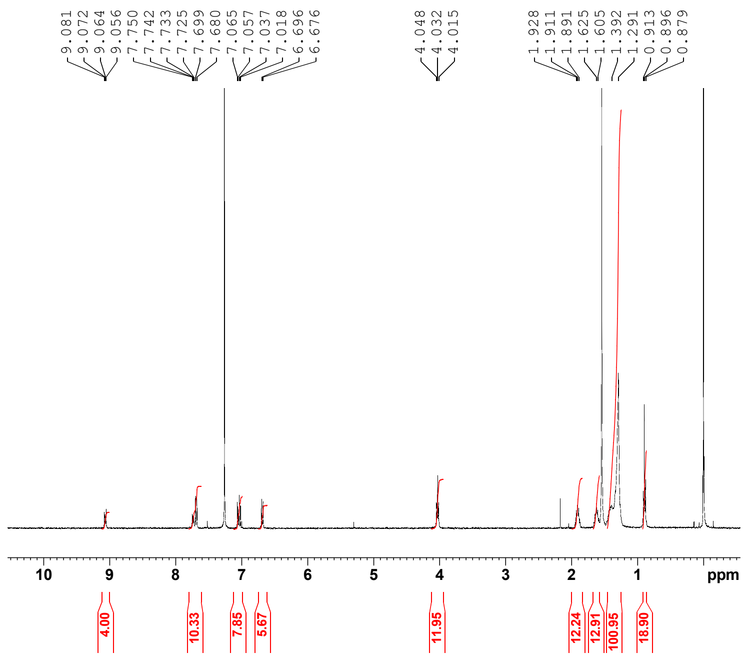

Fig. S17. Characterization of 2. ^{1}H NMR spectrum (400 MHz) of **2** in $CDCl_3$ at 25 °C.

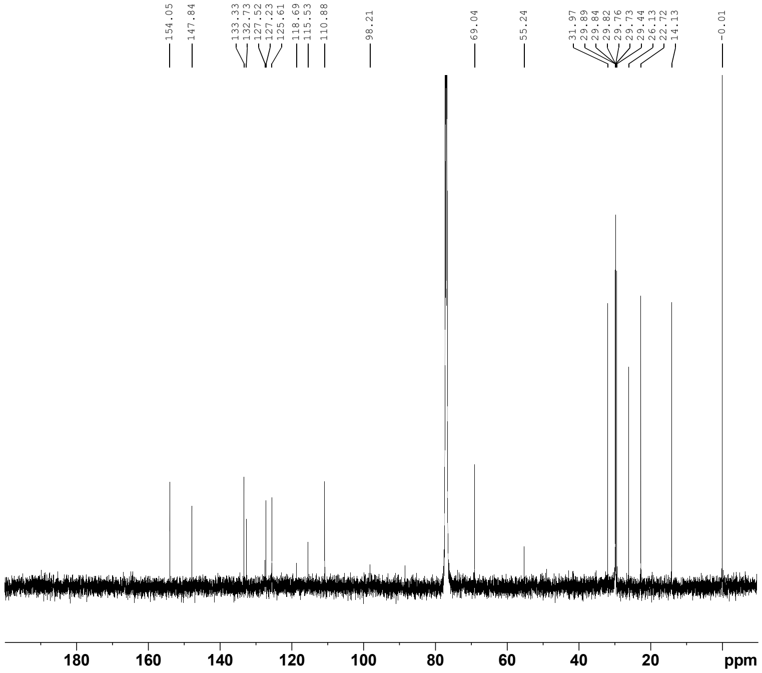

Fig. S18. Characterization of 2. ^{13}C NMR spectrum (126 MHz) of **2** in $CDCl_3$ at 25 °C.

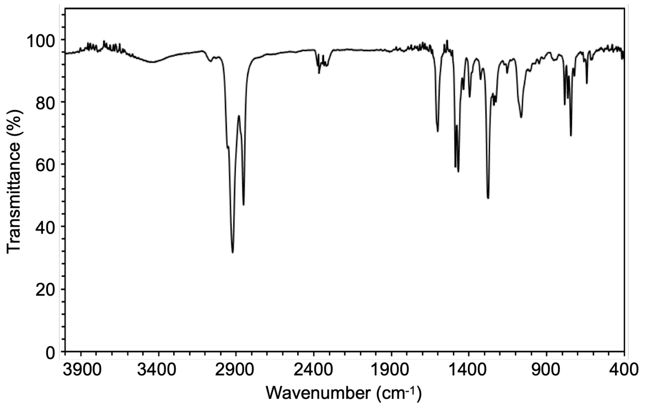

Fig. S19. Characterization of 2. FT-IR spectrum of **2** (KBr) at 25 °C.

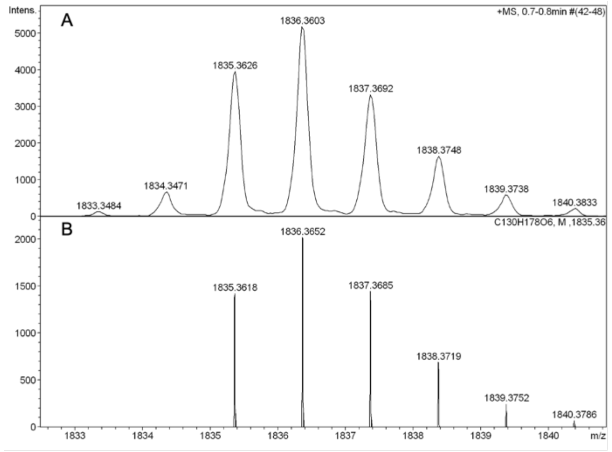

Fig. S20. Characterization of 2. (A) Observed and (B) simulated high-resolution APCI-TOF mass spectra of **2**.

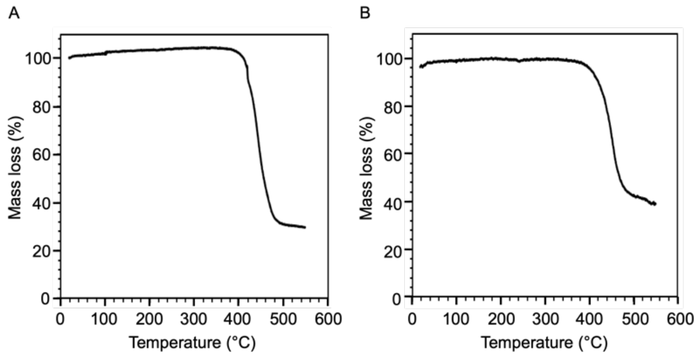

Fig. S21. Thermal stabilities of 1 and 2. TGA profiles of (A) **1** and (B) **2** measured at a scan rate of 10 °C/min under a nitrogen flow (50 mL/min).

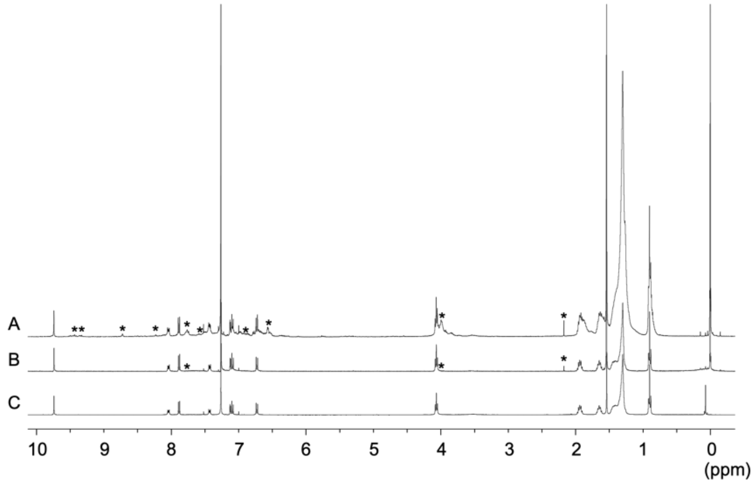

Fig. S22. Thermal stability of 1. ^{1}H NMR spectra (400 MHz, $CDCl_3$, 25 °C) of **1** after thermal treatment at (A) 230 °C, (B) 200 °C, and (C) 190 °C for 30 min under nitrogen. Signals arising from thermolysis products are marked using asterisks.

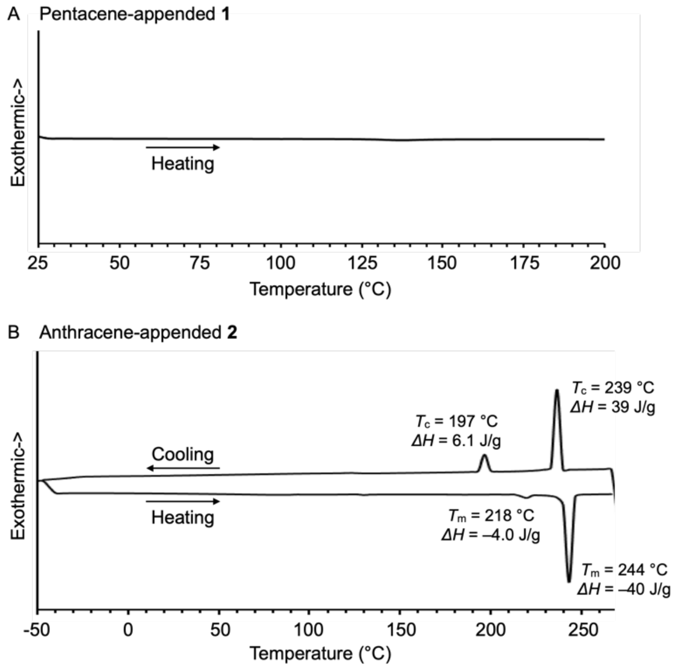

Fig. S23. Phase-transition behaviors of 1 and 2. DSC profiles of (A) **1** during a first heating and (B) **2** during a third heating/cooling cycle, measured at a scan rate of 10 °C/min under a nitrogen flow (50 mL/min).

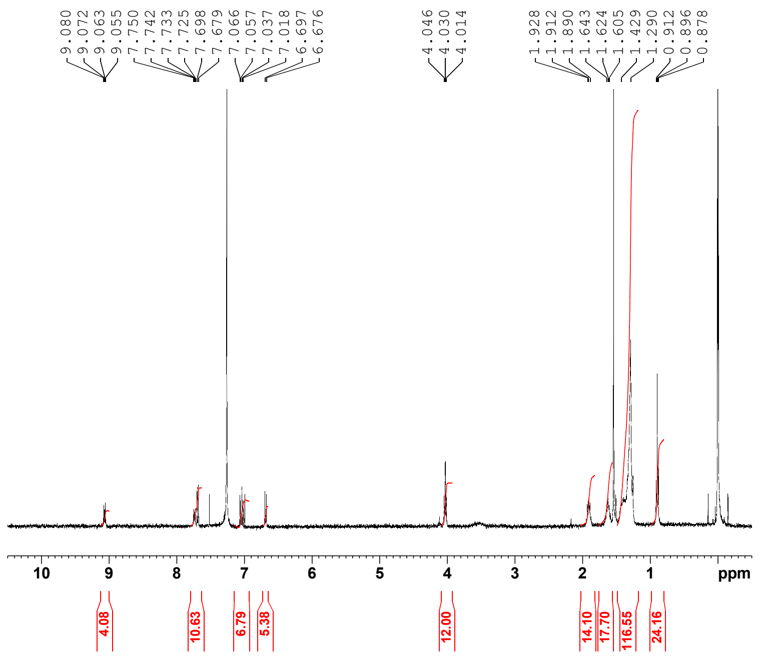

Fig. S24. Thermal stability of 2. ^{1}H NMR spectrum (400 MHz, $CDCl_3$, 25 °C) of **2** after thermal treatment at 270 °C for 30 min under nitrogen.

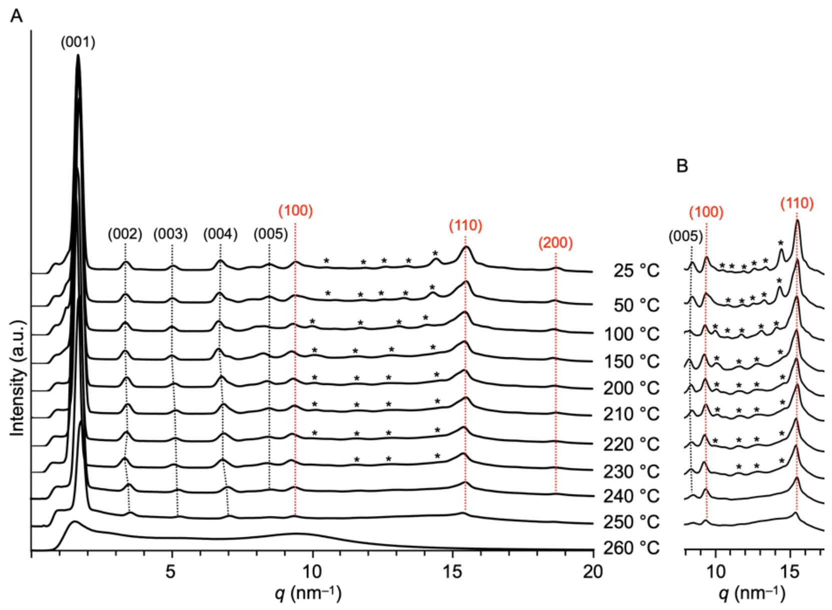

Fig. S25. Structural characterization of 2. (A) VT-PXRD patterns of a bulk solid sample of **2** upon heating at a rate of 10 °C/min and (B) their magnified views. Minor ill-defined diffractions are marked using asterisks.

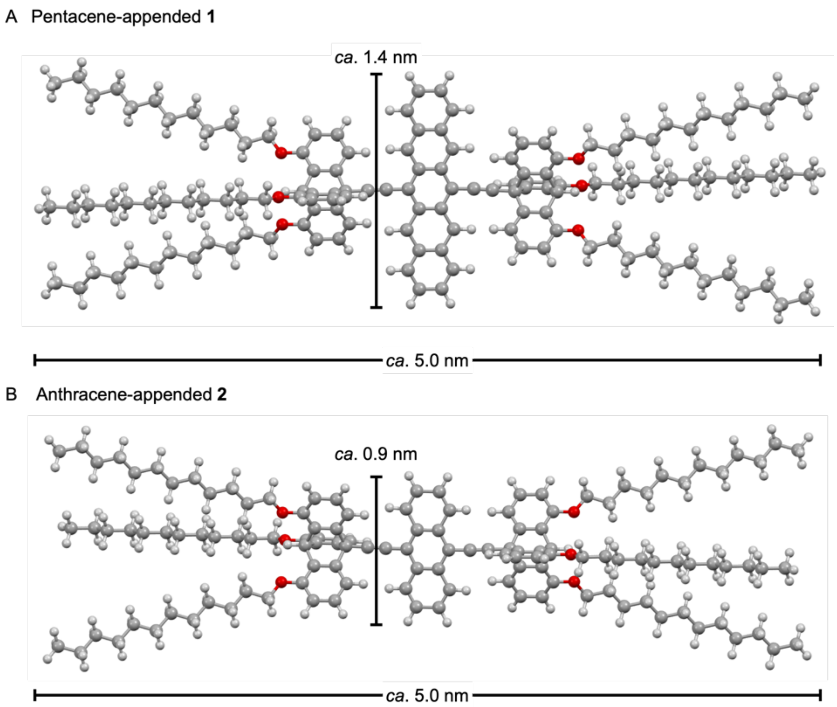

Fig. S26. Calculated structures of 1 and 2. Optimized structures of (A) **1** and (B) **2** obtained by molecular mechanics (MM2) calculations.

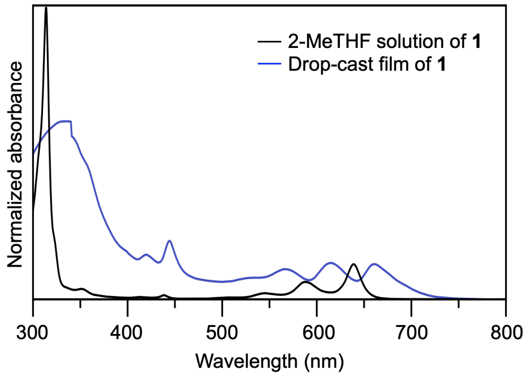

Fig. S27. Steady-state spectroscopic analysis of 1. Absorption spectra of a 2-MeTHF solution (5.0 μM) and a drop-cast film of **1** at 25 °C under air.

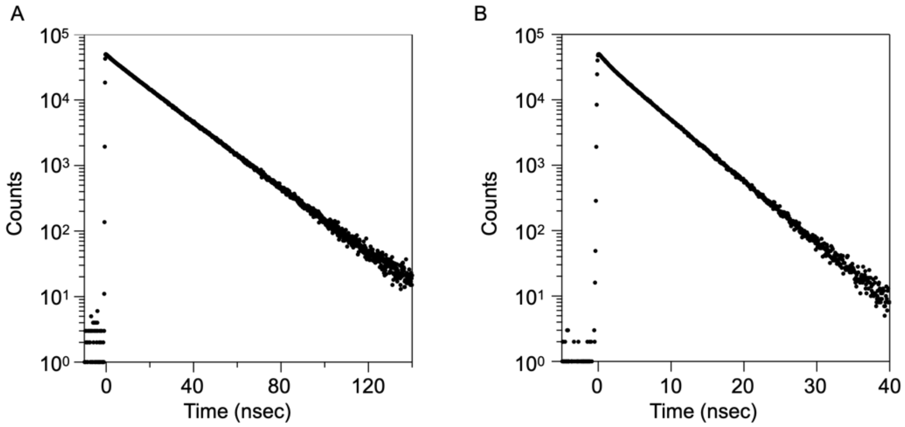

Fig. S28. Single photon counting analysis of the emission from 1 and 2. Emission decay profiles of (A) **1** (λ_{ex} = 585 nm) observed at 640 nm and (B) **2** (λ_{ex} = 404 nm) observed at 470 nm in 2-MeTHF (1.0 μM) at 25 °C under air.

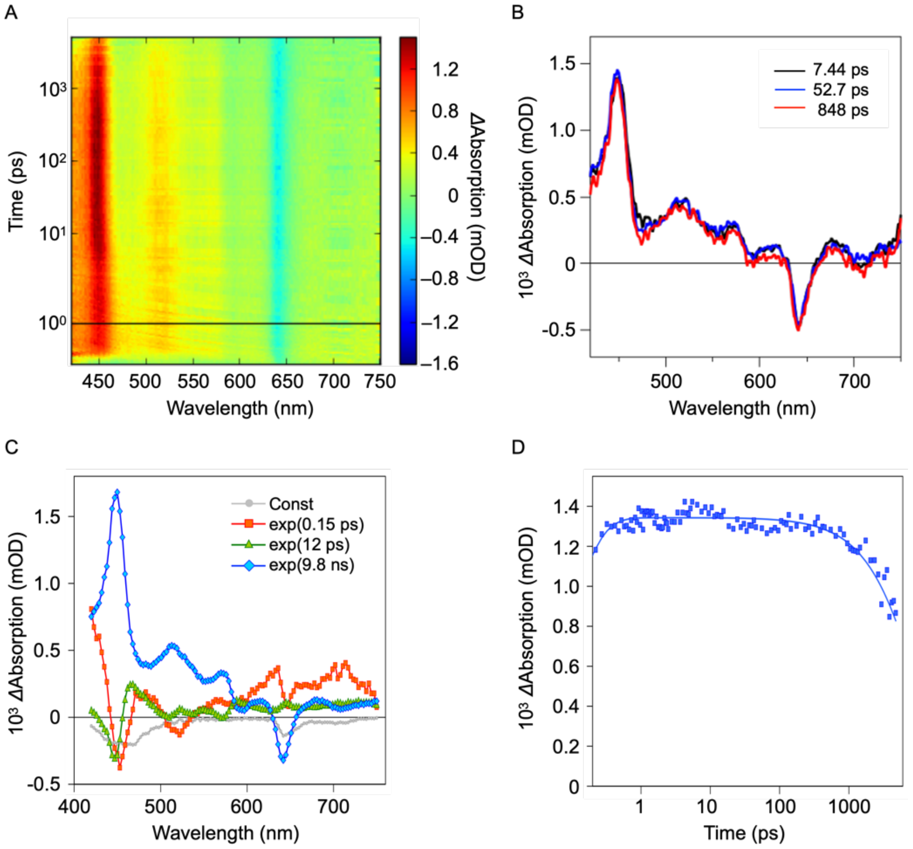

Fig. S29. Time-resolved spectroscopic analysis of 1. (A) 2D fs-TA spectra of **1** in 2-MeTHF (5.5 μM) at 25 °C (λ_{ex} = 360 nm, excitation density = 0.05 mJ/cm^2). (B) TA spectra of **1** at selected delay times taken from (A). (C) DAS of **1** in 2-MeTHF obtained from the global fitting analysis. (D) Decay profile of TA of a drop-cast film of **1** at 456 nm.

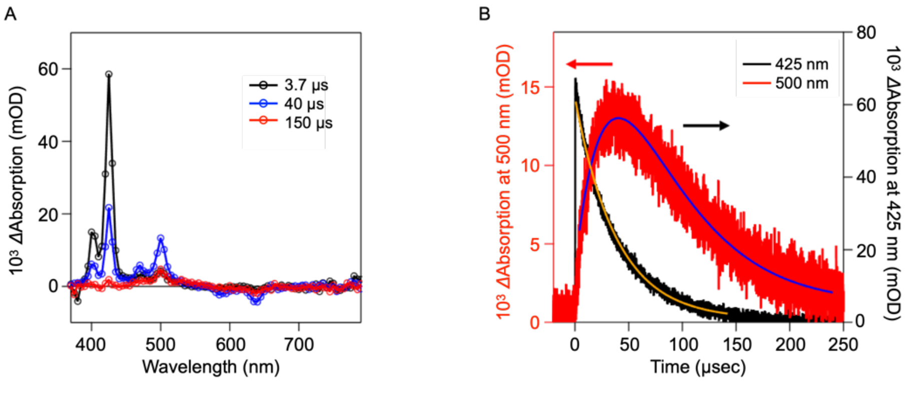

Fig. S30. Time-resolved spectroscopic analysis of 1. (A) 1D ns-TA spectra (λ_{ex} = 360 nm) of **1** in 2-MeTHF (5.5 μM) in the presence of anthracene (0.30 mM) at selected delay times. (B) Time-dependent TA changes at 425 nm (black) and 500 nm (red).

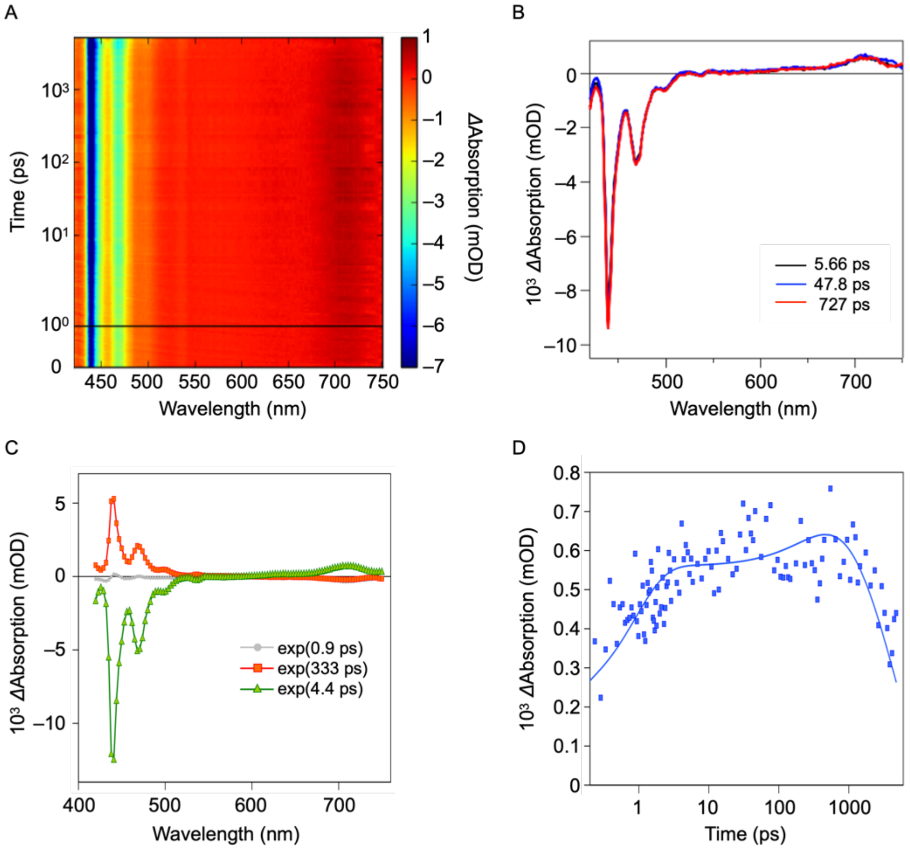

Fig. S31. Time-resolved spectroscopic analysis of 2. (A) 2D fs-TA spectra of **2** in 2-MeTHF (5.0 μM) at 25 °C (λ_{ex} = 400 nm, excitation density = 0.2 mJ/cm^2). (B) TA spectra of **2** at selected delay times taken from (A). (C) DAS of **2** in 2-MeTHF obtained from the global fitting analysis. (D) Decay profile of TA of a drop-cast film of **2** at 711 nm.

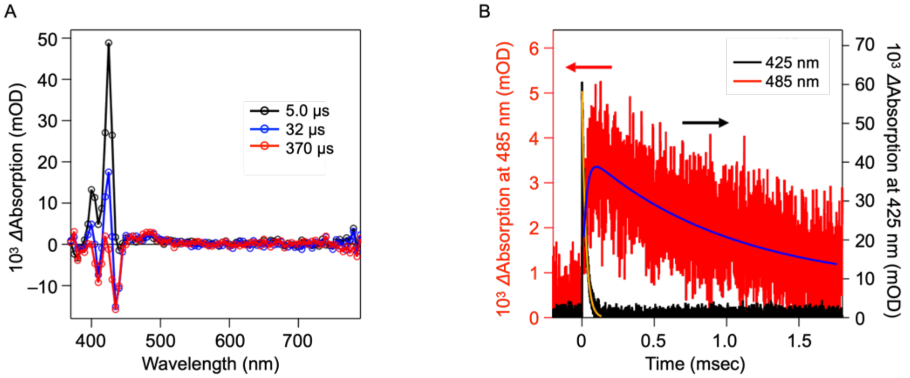

Fig. S32. Time-resolved spectroscopic analysis of 2. (A) 1D ns-TA spectra (λ_{ex} = 360 nm) of **2** in 2-MeTHF (5.0 μM) in the presence of anthracene (0.30 mM) at selected delay times. (B) Time-dependent TA changes at 425 nm (black) and 485 nm (red).

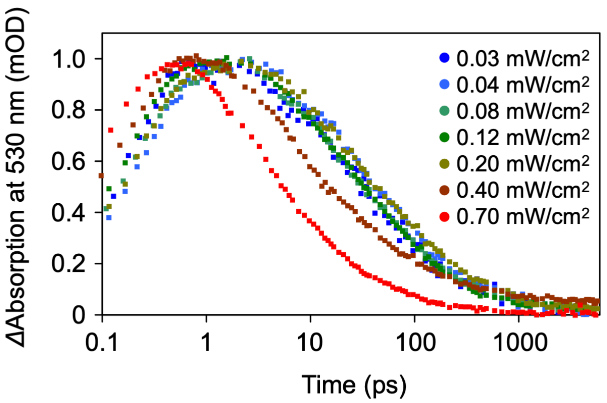

Fig. S33. Time-resolved spectroscopic analysis of 1. Decay profiles of the fs-TA of a drop-cast film of **1** at 530 nm, observed after laser-pulse excitation with different energy densities at 360 nm. There was no change in the decay profiles of a T-T absorption of the pentacene chromophore at 530 nm in the energy density range of 0.03 to 0.2 mJ/cm^2.

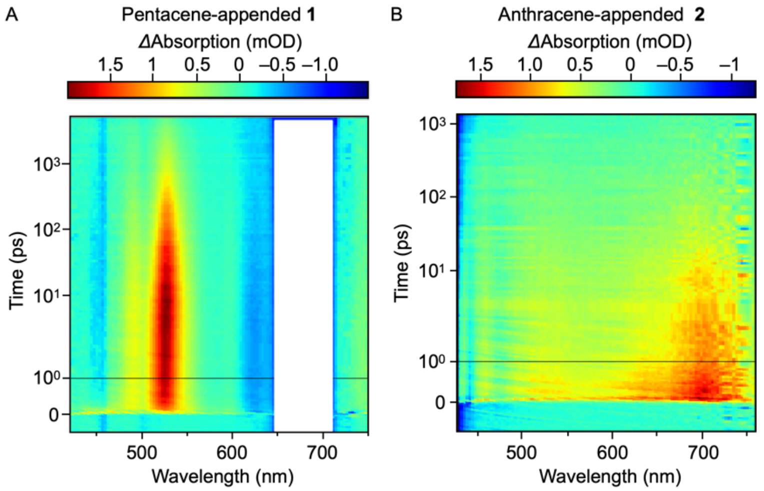

Fig. S34. Time-resolved spectroscopic analysis of 1 and 2. 2D fs-TA spectra of a drop-cast films of (A) **1** (λ_{Ex} = 670 nm) and (B) **2** (λ_{Ex} = 400 nm) on a quartz substrate. The time scale is linear till 1 ps delay (indicated by a horizontal black line) and logarithmic at longer delays. For (A), the white rectangle covers the scattering of the excitation pulse around 670 nm.

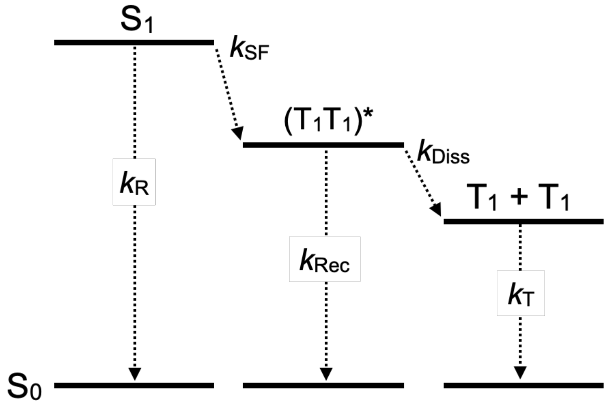

Fig. S35. Time-resolved spectroscopic analysis of 1. Schematic kinetic model involving S_1, $(T_1T_1)^*$, and T_1+T_1, used for global fitting analysis of the 2D fs-TA spectra of **1** (fig. S34A), where k_R, k_{SF}, k_{Rec}, k_{Diss}, and k_T are the rate constants for the $S_1 \rightarrow S_0$ transition, SF, $(T_1T_1)^* \rightarrow S_0$ transition, dissociation of $(T_1T_1)^*$ into T_1+T_1, and $T_1 \rightarrow S_0$ transition, respectively.

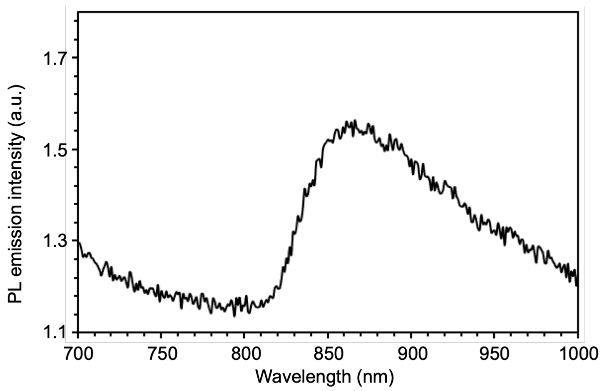

Fig. S36. Spectroscopic analysis of the phosphorescence from 2. Phosphorescence spectrum of **2** (1.0 μM) upon excitation at 410 nm in a glassy matrix of 2-MeTHF/iodomethane (9/1 v/v) at 77 K.

Table S1. Lifetimes of the excited-state chemical species of 1. The lifetimes (τ) for singlet excited state (S_1), a triplet pair [$(T_1T_1)^*$], and free triplets (T_1+T_1) of **1** obtained by global fitting analysis shown in Fig. 5.

	Lifetime (τ)
S_1	0.15±0.01 ps
$(T_1T_1)^*$	3±0.1 ps
T_1+T_1	240 ps

Table S2. Lifetimes of the excited-state chemical species of 2. The lifetimes (τ) for singlet excited state (S_1) and excimer of **2** obtained by global fitting analysis shown in Fig. 5.

	Lifetime (τ)
S_1	2.3 ps
Excimer	850 ps
